# Facilitative interspecific interactions in marine vertebrates across scales: from individuals to ecosystems

**DOI:** 10.1111/brv.70091

**Published:** 2025-10-20

**Authors:** Eduardo Döbber Vontobel, Sophie Smout, Jorge L. Rodrigues Filho, Ronaldo Angelini, Mauricio Cantor, Fábio G. Daura‐Jorge

**Affiliations:** ^1^ Department of Ecology and Zoology Federal University of Santa Catarina Córrego Grande Florianópolis SC 88037‐010 Brazil; ^2^ Sea Mammal Research Unit University of Saint Andrews Saint Andrews KY16 8LB UK; ^3^ Laboratory of Applied Ecology and Conservation, Department of Fisheries Engineering and Biological Sciences State University of Santa Catarina (UDESC/Laguna) Av. Cel Fernandes Martins, 270, Progresso Laguna Santa Catarina Brazil; ^4^ Environmental and Civil Engineering Department Federal University of Rio Grande do Norte Campus Universitario ‐ Lagoa Nova, CEP 59078‐970 Natal Rio Grande do Norte Brazil; ^5^ Oregon State University 2030 SE Marine Science Drive Newport OR 97365 USA

**Keywords:** apex predators, EBFM, ecosystem‐based fisheries management, ecosystem model, facilitation, mixed‐species groups, positive interaction

## Abstract

Facilitative interspecific interactions (FIIs) confer benefits to at least one participant without detriment to others. Although often less emphasised than antagonistic interactions in ecological studies, this review highlights the significant ecological role of FIIs across biological scales – from individual behaviours to population, community, and ecosystem‐level effects – with a focus on mobile marine vertebrates such as birds, mammals, and fish. These interactions enhance foraging success, shape predator–prey dynamics and contribute to the structure and function of marine ecosystems. FIIs include diverse associations such as multi‐species aggregations among marine apex predators (e.g. dolphins, seabirds, and surface‐feeding fish), mixed‐species shoals, fish cleaning mutualisms, and cooperative foraging involving predators, including humans. At the population level, FIIs can improve survival and fitness, impacting the life histories and population dynamics of marine apex predators, with some species exhibiting a clear dependence on heterospecific facilitation. Despite recent advances, gaps remain in our understanding of how FIIs scale up to influence marine communities and ecosystem processes, limiting their integration into management tools. Ecosystem models – often used to inform management decisions – typically focus on principles of resource flow and species interactions driven by predation and competition, often overlooking facilitation. Integrating FIIs into ecosystem modelling could enhance Ecosystem‐Based Fisheries Management, particularly for conserving vulnerable apex predators that may rely on facilitative interactions. Furthermore, FIIs involving humans and apex predators offer unique opportunities for data collection and model development, improving our understanding of the broader impacts of FII in marine environments, from individual behaviours to ecosystem functioning.

## I. INTRODUCTION

Facilitative interspecific interactions (FIIs), or positive interspecific interactions, occur when individuals of one species enhance the overall fitness of at least one participant without causing detriment to the other species (Stachowicz, 2001; Bruno, Stachowicz & Bertness, 2003; Bronstein, 2009). These interactions may significantly impact species distributions, population dynamics (Crowley & Cox, 2011), and community structure (Boucher, James & Keeler, 1982). The spectrum of FII is broad, ranging from casual and facultative associations to commensalism and obligate mutualisms (Bronstein, 1994, 2015; Bruno *et al*., 2003; Bulleri, 2009; Stachowicz, 2001), with such interactions being widespread across ecosystems. They are observed from the emergence of eukaryotic life forms (Boucher *et al*., 1982) to intricate relationships between flowers and seed‐dispersing pollinators (Baraza *et al*., 2007), complex coral reef networks (Stachowicz, 2001), and facilitative foraging among predators (Au & Pitman, 1986). Humans can also be involved, as in the cooperative foraging between honeyguide birds and honey hunters (Spottiswoode, Begg & Begg, 2016) or between wild dolphins and artisanal fishers (Simões‐Lopes, 1998; Cantor, Farine & Daura‐Jorge, 2023).

Historically, ecological research has prioritised antagonistic over facilitative interactions (Bruno *et al*., 2003). However, in a seminal essay, Gould (1988) shed light on Kropotkin's perspectives on ecological interactions. Drawing from the concept of “mutual aid” pioneered by the German zoologist K. F. Kessler, Kropotkin (1902) emphasised the significance of facilitative interactions, explicitly contrasting them with competitive dynamics. Departing from one of the central tenets of Darwin's *The Origin of Species*, both Kessler and Kropotkin challenged Darwin's emphasis on competition in the “struggle for existence”, arguing that cooperation could be equally, if not more, important (Todes, 1987). This viewpoint has gained traction with growing interest in the ecological roles of FII across diverse biological scales, from studies of individuals to impacts on whole ecosystems (Bruno *et al*., 2003; Bulleri, 2009; Ford & Roberts, 2019), encompassing plant–pollinator systems (Gotelli, Graves & Rahbeck, 2010), coral symbioses (Easson *et al*., 2014), and crustacean–fish partnerships (Vaughan *et al*., 2017).

These diverse examples highlight not only the ubiquity but also the variety of FIIs in form and function. To understand their ecological consequences, it is useful to distinguish among types of facilitation based on dependency and environmental context. Some species act as ecosystem engineers, physically altering habitats in ways that benefit others (Jones, Lawton & Shachak, 1994). In marine systems, facilitation is often facultative, where one species benefits from another but neither depends on the interaction for survival or reproduction. By contrast, obligate facilitation involves long‐term dependencies, where at least one species relies on the interaction to persist. Facultative interactions are particularly common in dynamic and open environments like the pelagic zone, where species distributions and behaviours often respond to fluctuating resources and conditions. Such associations can be highly context dependent, shifting along a continuum from facilitation to neutral or even antagonistic outcomes depending on ecological pressures (Bruno *et al*., 2003; Bulleri, 2009; Stachowicz, 2001).

Despite their now‐recognised ecological significance, FIIs have historically received less attention in the scientific literature due to perceptions that they are rare, difficult to demonstrate, or unimportant (Bronstein, 1994, 2009; Bruno *et al*., 2003). While interest in facilitation has increased, interactions involving highly mobile marine vertebrates remain relatively underexplored. Persistent inconsistencies in defining interactions across the facilitative spectrum (Hoeksema & Bruna, 2015) highlight the need for clear conceptual frameworks and functional glossaries to reduce ambiguity in ecological discourse (e.g. Silknetter *et al*., 2020; Cram *et al*., 2022; Crowley & Cox, 2011; Lang & Farine, 2017), particularly in the study of highly mobile organisms in the oceans. Moreover, within the spectrum of FIIs lies a dynamic array of strategies, each characterised by distinct definitions and subtleties. FII can also vary in terms of intentionality, ranging from incidental benefits (i.e. by‐products of behaviour directed at one's own fitness) to behaviours shaped by selection to benefit another species. For example, mobbing behaviour aimed at deterring predators may incidentally benefit multiple prey species, whereas coordinated foraging suggests mutual advantages selected over time. Although this review does not explicitly categorise FII by intentionality, we recognise that such distinctions are relevant to understanding their origins and stability (related terms are clarified in the glossary in Table 1).

**Table 1:** Glossary of terms related to facilitative interspecific interactions.

Facilitation and ecological interactions	
Commensalism	An ecological interaction in which one species benefits while the other experiences no significant positive or negative effect.
Facilitative interspecific interaction (FII)	An interaction between species where at least one benefits without causing harm to the other. These interactions can influence community structure, population dynamics, and ecosystem processes.
Facultative associations	Ecological interactions where the relationship between species is not essential for their survival or reproduction but still provides some benefit.
Facultative mutualism	A mutualistic relationship in which both species benefit but are not entirely dependent on each other for survival.
Mutualism	Any ecological interaction between two species in which both individuals experience a net benefit. Mutualistic relationships can involve resource exchange, protection, or other advantages that enhance survival or reproduction.
Obligate mutualism	A form of mutualism where both species strictly depend on each other for survival or reproduction.
Positive interaction	An ecological interaction where at least one species benefits, including mutualism, commensalism, and facilitation.
Strategies and facilitative behaviours	
Aggregation	The co‐occurrence of two or more individuals of the same or different species in space and time due to a shared environmental driver, without necessarily involving social or cooperative interactions.
Cooperative foraging	A type of cooperative hunting where individuals from the same or different species coordinate their actions to increase hunting success.
Follower–nuclear species	A behavioural interaction in which one species (nuclear) is actively followed by another (follower) that benefits from access to resources, information, or protection.
Kleptoparasitism	A feeding strategy in which an individual steals food or prey that another individual has captured or is actively hunting.
Local enhancement	A behavioural phenomenon in which an individual is attracted to a location based on the presence or activity of another, usually related to foraging or predator avoidance.
Mixed‐species feeding association (MSFA)	A specific type of *mixed‐species group* where individuals from different species gather to forage together, often benefiting from increased foraging efficiency or reduced predation risk.
Mixed‐species group (MSG)	Temporary or long‐term group formed by individuals of different species interacting or coexisting within the same ecological community or habitat. These groups may arise for foraging benefits, predator avoidance, social interaction, or mutualistic relationships.
Social predation	A form of cooperative hunting where predators work together to locate, capture, and consume prey, improving hunting success and efficiency.
Key species and functional roles	
Ecosystem engineer species	Species that actively modify their environment, creating, maintaining, or altering habitats in ways that benefit other species. These modifications can be physical (e.g. beavers building dams, corals forming reefs) or biogeochemical (e.g. earthworms altering soil composition).
Flagship ecological interaction	A rare, culturally significant, and ecologically relevant interaction between two species, where conserving the interaction itself has broader ecological and social benefits.
Keystone facilitator	A species critical in facilitating interactions between other species, significantly influencing community structure and biodiversity.
Keystone species	A species that has a disproportionately large impact on its ecosystem relative to its abundance. Keystone species regulate community structure by affecting species interactions such as predation, competition, and facilitation.
Structuring species	Species that provide habitat complexity and stability, influencing ecosystem composition and organisation. Unlike ecosystem engineers, structuring species do not necessarily modify their environment but create physical conditions that support other organisms (e.g. kelp forests, mangroves, and mussel beds).

In this review, we explore how FIIs among marine vertebrates manifest and propagate across biological scales, from individual behaviours to population processes, community structure, and ecosystem‐level effects. This cross‐scale framing allows us to consider not only the direct benefits between species but also the broader ecological consequences that emerge over space and time. To contextualise, our synthesis draws from species coexistence theory (Chesson, 2000), mutualism ecology (Bronstein, 1994), and ecological network theory (Kéfi *et al*., 2016). We focus primarily on direct facilitative interactions – behavioural coordination, spatial association, and resource provisioning (e.g. cooperative foraging and cleaning symbioses) – as these are more readily documented in highly mobile species in marine environments. Indirect effects, such as those mediated through habitat modification or food‐web dynamics (Thomsen *et al*., 2010; Bruno *et al*., 2003; Bulleri *et al*., 2016), lie beyond the scope of this review but warrant future analyses.

## II. ANIMAL GROUPS UNDER ANALYSIS

**Fig. 1:**
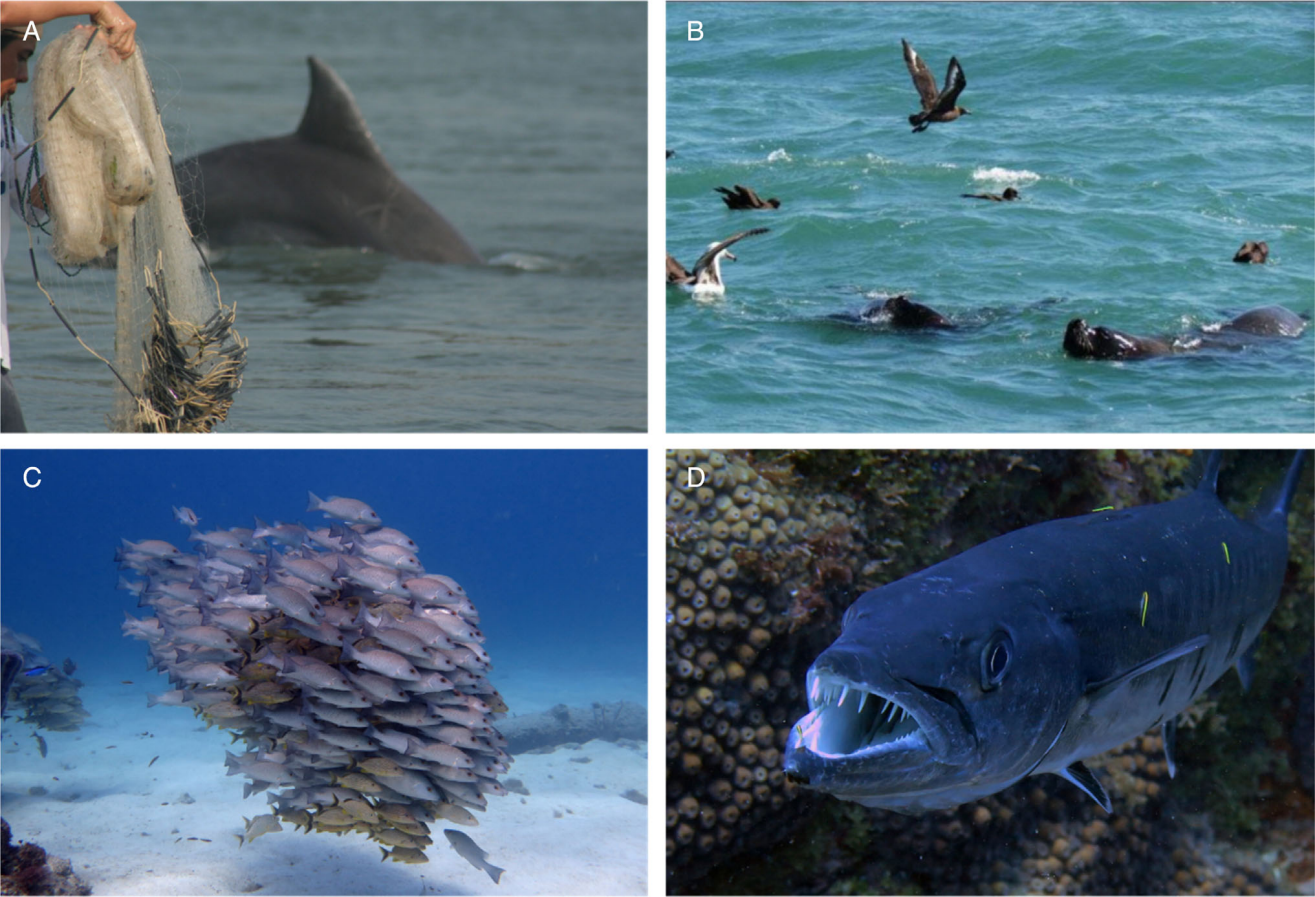
Exemplar facilitative interspecific interactions among marine vertebrates. (A) Cooperative fisheries between net‐casting fishers and *Tursiops truncatus gephyreus* in Laguna, southern Brazil, where dolphins actively herd fish toward fishers' nets, increasing capture success for both fishers and dolphins (source: F.G. Daura‐Jorge). (B) Procellariiform seabirds foraging in association with subsurface pinnipeds in Tramandaí, southern Brazil (source: G. Frainer), where pinnipeds drive prey towards the surface, making them available to seabirds. (C) A joint school of fish from the genera *Lutjanus* and *Haemulon* in Cozumel, Mexican Caribbean, where aggregating in a multispecies school enhances predator avoidance and foraging efficiency. (D) *Sphyraena barracuda* being cleaned by *Elacatinus phthirophagus* in Fernando de Noronha, Brazil, where the goby removes ectoparasites and dead tissue, benefiting the barracuda while gaining food (source: S.R. Floeter).

Despite numerous studies documenting FIIs among marine fish (Strand, 1988; Caves, 2021a,b) and marine apex predators (Au & Pitman, 1986; Veit & Harrison, 2017; Fig. 1), it remains unclear how these interactions impact patterns and processes at various biological scales, including their effects on resultant ecosystem services utilised by humans. Marine fish, for instance, are critical resources (Food and Agriculture Organization, 2022; Holmlund & Hammer, 1999). Understanding how FIIs shape their populations and behaviours is vital, as they provide food, support subsistence livelihoods, and underpin the socio‐economic sustainability of fishing communities. Consequently, they are key targets in Ecosystem‐Based Fisheries Management (EBFM; Link, 2010).

Similarly, marine apex predators also play essential roles in regulating marine food webs, impacting species targeted by industrial fishing (e.g. tunas and sharks; Ferretti *et al*., 2010) and those important to small‐scale subsistence fisheries [e.g. pinnipeds (Hurtubise, 2016; Jensen, Sheehan & MacLean, 2009)]. Clarifying the role of FIIs in their ecology is therefore critical to understanding food‐web structure and dynamics. Moreover, many apex predators such as seabirds and cetaceans are central to EBFM strategies aligned with international biodiversity agreements, such as the Convention on Migratory Species (CMS; https://www.cms.int) and the Convention on International Trade in Endangered Species (CITES; https://cites.org). Understanding how FII influences their life histories and population dynamics is thus crucial for effective conservation planning. In the following sections, we review FII across biological scales, focusing specifically on marine fish, seabirds, and cetaceans, emphasising apex predators. We conclude by summarising key insights that can guide marine species conservation and by identifying future research avenues to address current knowledge gaps.

## III. INDIVIDUAL‐LEVEL FACILITATIVE INTERSPECIES INTERACTIONS

Facilitation at the individual level underpins broader ecological effects, often emerging through behavioural coordination, local enhancement, or social learning. Drawing on niche theory and species coexistence frameworks, which recognise that interspecific behaviours – from incidental associations to structured cooperation – can influence survival, resource use, and fitness, we review cases where facilitation shapes individual behaviour with potential cascading consequences across populations, communities, and ecosystems.

**Fig. 2:**
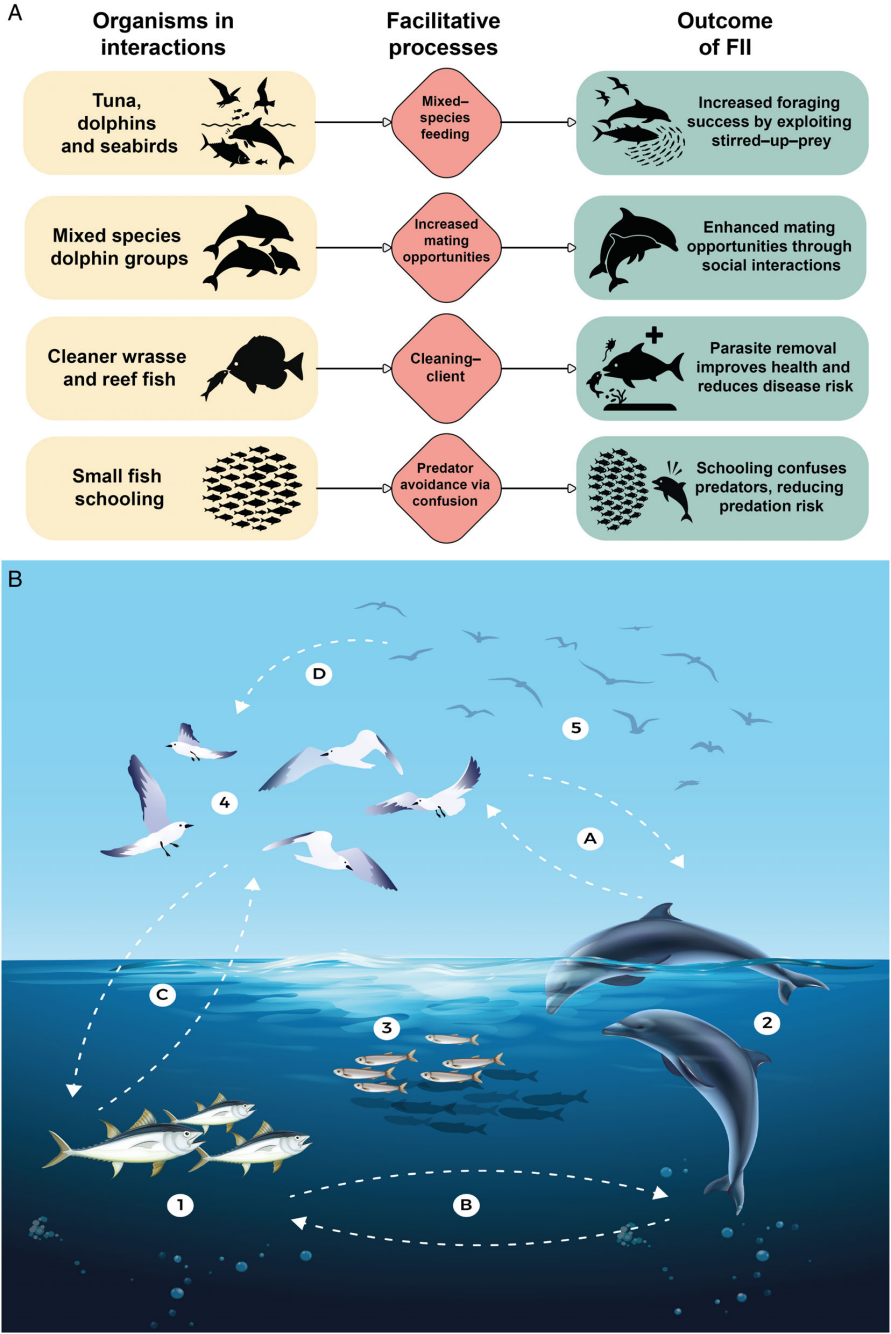
Diversity and complexity of facilitative interspecific interactions (FIIs) in marine ecosystems. (A) Summary of different types of facilitative interactions, showing example taxa, interaction types, and ecological outcomes. These include feeding, mating, cleaning, and predator avoidance, each contributing to survival, reproduction, and ecosystem balance. (B) Visual representation of facilitative foraging interactions among pelagic vertebrates. Numbers represent functional groups: (1) predatory fish (e.g. tuna); (2) marine mammals (e.g. dolphins); (3) forage fish (e.g. sardines); (4) predatory seabirds (e.g. terns); (5) kleptoparasitic seabirds (e.g. frigatebirds). Letters indicate interactions: (A) dolphins and seabirds; (B) dolphins and tuna; (C) seabirds and tuna –which may range from mutualism to competition; (D) local enhancement/follower–nuclear relationships.

Numerous studies have documented marine apex predators engaging in potential FII with other marine predators (Fig. 2). For example, Bearzi (2006) described behavioural correlations between California sea lions (*Zalophus californianus*) and three dolphin species (*Tursiops truncatus*, *Delphinus delphis*, and *D. capensis*) during social predation, including diving, travelling, surface‐feeding, and socialising. These correlations were stronger during aggregation events than in short‐term associations, suggesting foraging advantages for individual sea lions. However, dolphin behaviour ranged from cooperative strategies (Gallo‐Reynoso, 1991) to kleptoparasitism and commensalism (Bearzi, 2006), where dolphins opportunistically steal prey or benefit from other species' foraging without direct impact. This illustrates a gradient of interspecific interactions, from antagonistic to neutral or weakly positive facilitation, depending on the ecological context and mutual benefit.

Many studies have also evaluated the impact of FII on cetaceans within mixed‐species groups (MSGs) (see Stensland, Angerbjörn & Berggren, 2003; Fig. 2A). Such groups typically consist of individuals seeking advantages through facilitative interactions, potentially improving foraging success or predation protection. Additional benefits include increased chances of reproduction and survival, as well as opportunities for mating practice, playful behaviour, and alloparental care. MSGs may offer advantages over single‐species groups by reducing breeding competition (Dunbar, 1993; Buchanan‐Smith, 1999). These overlapping benefits (Carlson *et al*., 2023; Goodale, Beauchamp & Ruxton, 2017; Stensland *et al*., 2003) exemplify FII. For instance, Herzing & Johnson (1997) and Elliser & Herzing (2016) documented affiliative and aggressive behaviours between Atlantic spotted dolphins (*Stenella frontalis*) and bottlenose dolphins (*Tursiops truncatus*) in MSGs in the Bahamas. Similarly, Syme, Kiszka & Parra (2021) observed common socialising and travelling in MSGs of Australian humpback dolphins (*Sousa sahulensis*) and Indo‐Pacific bottlenose dolphins (*Tursiops aduncus*), with foraging observed in approximately 11% of sightings. They proposed a primary social function for these MSGs, providing social learning and mating opportunities, with antipredator behaviour being secondary. Other studies have documented the frequency of MSGs compared to single‐species groups (Syme, Kiszka & Parra, 2023), highlighting their importance for certain species (Acevedo‐Gutiérrez, 1999; Thompson, 2010; Kiszka *et al*., 2011).

Cetaceans also frequently interact with seabirds (Evans, 1982), often near predatory fish (Ballance, Pitman & Fiedler, 2006; Clua & Grosvalet, 2001; Veit & Harrison, 2017; Rogan & Mackey, 2007; Guse *et al*., 2013; Goyert, Manne & Veit, 2014). These interactions typically involve a triadic dynamic where subsurface predators (tunas, dolphins, and whales) corral fish towards the surface, forming prey balls that aid capture for all involved (Au & Pitman, 1988; Veit & Harrison, 2017; Fig. 2). Documented cases include sharks trailing tunas (Au, 1991) and numerous seabird–tuna interactions (Au & Pitman, 1988; Hebshi, Duffy & Hyrenbach, 2008). These range from antagonistic competition and parasitism to facilitative scenarios (Veit & Harrison, 2017; Bronstein, 1994; Stachowicz, 2001) (Fig. 2). Total dependence occurs in some cases, such as between Parkinson's petrels (*Procellaria parkinsoni*) and melon‐headed (*Peponocephala electra*) and false killer whales (*Pseudorca crassidens*) (Pitman & Ballance, 1992). Evans (1982) also details seabird–dolphin associational behaviours, including mass attacks (Clua & Grosvalet, 2001) and prey disruption (Thiebault *et al*., 2016), which may alleviate foraging challenges and constitute FII (Ashmole & Ashmole, 1967; Au & Pitman, 1986; Ballance *et al*., 2006; Guse *et al*., 2013). These temporary or obligate interspecific associations are thought to enhance individual predator fitness (Pitman & Ballance, 1992). However, studying these complex interactions faces methodological challenges inherent in underwater research (Norris & Dohl, 1980; Scott *et al*., 2012), although recent advancements in acoustics (Ceyrac *et al*., 2018; McInnes *et al*., 2024) and biologging (Beltran *et al*., 2024; Clayton *et al*., 2023; Hessing *et al*., 2024) have facilitated progress.

While some facilitative interactions involve large, dynamic aggregations, others are characterised by more direct and structured cooperation, where individuals modulate behaviour for shared goals or mutual benefits (Chase, 1980; Clutton‐Brock, 2009; Dugatkin, 2002; Bronstein & Sridhar, 2024). Such cooperative behaviours include group hunting (Dugatkin, 2002; Bailey, Myatt & Wilson, 2013), cooperative kinship (Smith, 2014; Dugatkin, 2002), cooperative breeding (Hatchwell, 2009), and joint defence (Dugatkin, 2002; Hager & Jones, 2009). A fascinating example is cooperative fishing involving net‐casting fishers and dolphins (Simões‐Lopes, 1998) coordinating to access mullet (*Mugil* spp.) (Cantor *et al*., 2023; Fig. 1A). Lang & Farine (2017) analysed similar social predation cases, proposing a multidimensional framework for examining this form of interaction considering communication, sociality, and level of dependency, highlighting the intricate nature of FIIs among marine apex predators, including dolphins (Hebshi *et al*., 2008; Au & Pitman, 1988) and fish (Bshary *et al*., 2006).

A common manifestation of these dynamics is local enhancement, where apex predators adaptively use information from conspecifics or other species for foraging (Pöysä, 1992; Buckley, 1996; Fig. 1B), often fitting the follower–nuclear species perspective (Inagaki *et al*., 2020). This behaviour has been observed across various marine taxa: seabirds [including Procellariiformes (Thiebault *et al*., 2014) and penguins (Silverman *et al*., 2004)] as well as cetaceans (Veit & Harrison, 2017), pinnipeds (Bearzi, 2006) and fish [including bony fish (Strand, 1988) and sharks (White *et al*., 2022)], all of which have been seen following other species to locate prey. These interactions often involve a nuclear species (leader) and follower species exploiting social information for foraging. While followers clearly benefit, nuclear species may also gain advantages like improved prey capture through mass attacks or group foraging disruption. Yet, assessing the magnitude and direction of these benefits remains challenging.

A crucial conceptual challenge is distinguishing true facilitation from basic functional complementarity or accidental co‐occurrence. For instance, dolphin, tuna, and seabird aggregations targeting schooling prey may result from independent responses to shared cues like prey density, lacking active interspecific coordination. Our framework defines facilitation requiring at least one species consistently benefiting from the presence or actions of another beyond chance. This benefit may manifest as improved prey accessibility, reduced search time, or enhanced predator avoidance. While some multi‐species foraging groups may arise from incidental convergence (i.e. non‐intentional co‐occurrence) that leads to facilitative outcomes, others show behavioural adjustments or learning that qualify as truly facilitative.

This continuum is also evident in interspecific mobbing, where one species assists others in deterring predators. For example, humpback whales (*Megaptera novaeangliae*) defend conspecifics and other species like gray whales (*Eschrichtius robustus*), Weddell seals (*Leptonychotes weddellii*), and sea lions from killer whale (*Orcinus orca*) attacks, an antipredator strategy (Pitman *et al*., 2017) The motivations behind these interspecific responses remain puzzling, possibly reflecting predator harassment culture or generalised protective behaviour triggered by distress cues. Pitman *et al*. (2017) suggest that future research should explore whether these responses arise from social learning or innate tendencies shaped by cultural or natural selection. Other examples of marine predator mobbing include Galápagos fur seals (*Arctocephalus galapagoensis*), Galápagos sea lions (*Zalophus wollebaeki*), and Australian fur seals (*Arctocephalus pusillus doriferus*) mobbing sharks (Barlow, 1972; Trillmich, 1996; Dickie, 2005), Steller sea lions mobbing killer whales (Heise *et al*., 2003), and dolphins mobbing sharks (Essapian, 1953; Connor, 2000).

While the previous examples highlighted interspecific coordination in predator deterrence, similar dynamics occur in foraging. Traditionally, fish behavioural studies focused on single‐species shoals (Norris & Schilt, 1988; Pitcher, 2012). Recent research, however, has examined multi‐species shoals, particularly among coral‐reef species (Lukoschek & McCormick, 2000; Strand, 1988; Fig. 1C). Ormond (1980) categorised four potentially facilitative multi‐species foraging strategies: (1) following and scavenging, where large carnivorous fish are trailed by other carnivores and opportunistic feeders; (2) hunting by riding, where a predator positions itself near another species to conserve energy; (3) interspecific joint hunting, involving cooperation between predators; and (4) aggressive mimicry, where a predator mimics a harmless species. By contrast, non‐predatory species such as scarids and acanthurids, which primarily graze on algae or bite sessile corals, appear to benefit from group foraging through social facilitation or local enhancement. Studies have shown that bite rates in these non‐predatory species correlate with shoal size (Baird, 1993), and individuals in larger shoals feed for longer periods (Foster, 1985; Wolf, 1987). These benefits often arise through mechanisms such as local enhancement from a nuclear species (Lukoschek & McCormick, 2000) and social facilitation among attendants is also well documented, as seen between the puddingwife wrasse (*Halichoeres radiatus*) as a nuclear species and the bar jack (*Caranx ruber*) as an attendant (Baird, 1993).

Many associations, driven by foraging, also reduce predation risk. Mixed‐species shoals may enhance collective vigilance, as individuals respond to others' alarm cues or escape behaviours (Fig. 1C). Confusion effects also arise from different morphologies and escape tactics. Sazima *et al*. (2007) describe the goatfish (*Pseudupeneus maculatus*) as a nuclear species in multi‐species foraging groups, attracting jacks and other followers. Primarily foraging‐centred, these aggregations also offer antipredator advantages by increasing detection and reducing individual risk, suggesting that multi‐species shoals are flexible strategies balancing feeding efficiency and predator avoidance. Foraging benefits enhancing fitness can also arise from socially acquired information (Gil *et al*., 2017), evident in collective behaviours across taxa, including ontogenetic changes in fish within subtropical inshore habitats. Juvenile fish exchange information in MSGs, using behavioural cues from species they will later compete with as adults. This highlights the role of FIIs in shaping ontogenetic development, suggesting potential commensal or mutualistic dynamics within juvenile fish MSGs (Haak *et al*., 2020). Such intricate foraging associations are not limited to juveniles. A notable example in adults is the cooperative hunting between grouper (*Plectropomus pessuliferus*) and giant moray eel (*Gymnothorax javanicus*), where visual signals from the grouper coordinate behaviour, enhancing foraging success over solitary hunting (Bshary *et al*., 2006).

Beyond foraging‐related facilitations, fish engage in other notable FIIs such as cleaning interactions where cleaner fish remove ectoparasites and debris from larger ‘client’ fish, which benefit from parasite reduction (Beebe, 1928) (Figs 1D and 2A). Widely cited as a ‘textbook’ mutualism (Caves, 2021a) and sometimes cooperative (Limbaugh, 1961; Feder, 1966), these interactions involve clients modifying behaviour based on cleaning needs (Caves, 2021a) and signalling for coordination. Importantly, cleaning mutualisms are among the few marine interspecific interactions with experimentally demonstrated fitness benefits (Grutter, 1999; Bshary, 2003), showing increased client recruitment, survival, and local biodiversity. Soares *et al*. (2012) also found that clients adjust cleaning behaviour based on prior interactions, suggesting a learned component.

However, cleaning interactions also illustrate the context‐dependent nature of interspecific outcomes, transitioning from mutualistic to parasitic based on partner behaviour, environmental pressures, or individual decisions, a classic mutualism–parasitism continuum. While clients typically benefit from parasite removal, cleaners sometimes cheat by removing scales, mucus, or tissue (Limbaugh, 1961; Grutter, 1997; Soares *et al*., 2008a,b; Quimbayo *et al*., 2023). This imposes costs on clients and introduces conflict. Such variability highlights the complexity of cleaning interactions, where net outcomes are shaped by partner behaviour, ecological conditions, and individual decision‐making (Bronstein, 1994). Although generally facultative, marine cleaning mutualisms are mediated by signalling cues that influence engagement. Despite decades of research, key questions remain about abiotic, ecological, social, behavioural, and genetic factors affecting their dynamics and stability (Soares *et al*., 2008a,b; Caves, 2021a,b). Thus, cleaner stations remain valuable model systems for understanding the costs, benefits, and plasticity of interspecific facilitation in marine ecosystems.

## IV. POPULATION‐LEVEL CONSEQUENCES OF FACILITATIVE INTERSPECIES INTERACTIONS

Facilitation between species can enhance individual fitness by improving vital rates such as survival and fecundity, thus boosting population growth rates (Bronstein, 1994; Bruno *et al*., 2003; Crowley & Cox, 2011; Svenning *et al*., 2014). However, traditional population ecology has largely concentrated on antagonistic, density‐dependent factors like resource competition, disease transmission, and predation (Bronstein, 1994), often neglecting the vital role of facilitative interactions in marine ecosystems (Bruno *et al*., 2003) and their evolutionary implications (Stensland *et al*., 2003; Goodale *et al*., 2017).

Seabirds, once considered transient participants in ephemeral feeding aggregations with minimal population and community impact (Munn & Terborgh, 1979; Sridhar, Beauchamp & Shanker, 2009), are now recognised for their significant roles in interspecific foraging associations (Veit & Harrison, 2017), with clear population‐level consequences of FII. For instance, species such as the sooty tern (*Sterna fuscata*) and great frigatebird (*Fregata minor*) rely heavily on tuna and dolphins to access surface prey (Brewer & Hertel, 2007). Other aerial predators, such as frigatebirds themselves, even exploit prey like flying fish that evolved aerial escape tactics to avoid marine predators, only to become vulnerable to capture in the air. This dependence on the facilitator implies that fluctuations in the abundance or behaviour of these predators can cascade through seabird foraging efficiency, breeding success, and ultimately, population dynamics.

Although FIIs among seabirds are prevalent, with benefits generally outweighing antagonistic costs (Veit & Harrison, 2017), the underlying mechanisms driving these interactions remain elusive. A study on Aride Island in the Seychelles documented that roseate tern (*Sterna dougallii*) reproductive success improved through associations with predatory fish, which lowered breeding failure rates by increasing chick prey availability, demonstrating significant benefits from facilitative interactions with typical competitors (Ramos, 2000). Alongside other predators like tuna, dolphins, marlins, and billfish, seabirds use multidimensional predatory strategies to disrupt fish schools, boosting prey capture efficiency and thereby enhancing their foraging success and population resilience (Lett *et al*., 2014; Thiebault *et al*., 2016).

FIIs can directly influence population dynamics and individual variation within species. In southern Brazil, Bezamat *et al*. (2018) examined how cooperation between Lahille's dolphins (*T. truncatus gephyreus*) and net‐casting fishers was linked to individual variation and population dynamics, providing a framework to assess the demographic consequences of FII. While fishers benefit through increased catch rates (Simões‐Lopes, 1998; Cantor *et al*., 2023), long‐term effects on dolphins also appear positive, with higher survival rates for cooperating individuals (Bezamat *et al*., 2018). Similarly, Syme *et al*. (2023) explored how MSGs involving Australian humpback dolphins (*Sousa sahulensis*) and Indo‐Pacific bottlenose dolphins (*Tursiops aduncus*) in Northwest Cape, Western Australia, serve social functions, such as refining social skills and providing alloparental care, alongside antipredator benefits. Conversely, population density and composition (e.g. relative abundance of adults and juveniles) can also shape FIIs by influencing how individuals engage with conspecifics and heterospecifics across space and time. This highlights how population characteristics can mediate facilitative interactions. For instance, Elliser & Herzing (2016) demonstrated that population characteristics influenced the frequency and nature of mixed‐species encounters between Atlantic spotted dolphins (*Stenella frontalis*) and Atlantic bottlenose dolphins (*Tursiop truncatus*) in the Bahamas, suggesting a role of demographic structure in maintaining interspecific associations and overall population stability.

These population‐level consequences of FIIs are not limited to seabirds and marine mammals. Similar patterns emerge in other taxa, including fish, where facilitation influences survival and population dynamics during critical life stages. Juvenile fish use social information in FIIs, including visual and chemical cues about predation risk and food availability (Haak *et al*., 2020). In mixed‐species prey guilds of similarly sized individuals, this information sharing enhances collective vigilance, coordinated escape responses, and detection of food patches (Paijmans, Reardon & Ward, 2020; Mukherjee & Bhat, 2023; Goodale *et al*., 2010). These mechanisms improve survival and foraging efficiency during vulnerable early developmental stages, even though many species later become competitors as adults (Lukoschek & McCormick, 2000). Such facilitative dynamics can also contribute to the stability of prey communities (Mukherjee & Bhat, 2023). Beyond prey guilds, empirical studies of cleaning interactions on coral reefs further underscore the importance of FIIs. For example, Waldie *et al*. (2011) demonstrated that removing cleaner fish led to declines in species abundance, growth, survival, and recruitment, highlighting the foundational role of such interactions in maintaining ecological balance.

However, capturing these complex dynamics in predictive models remains a significant challenge. Although mutualistic population dynamic models, such as those adapting the Lotka–Volterra framework by Gause & Witt (1935), exist, they often face mathematical instability, leading to unrealistic, unbounded growth – a problem identified in the work of Gause & Witt (1935) and addressed by Holland, DeAngelis & Bronstein (2002). Stable coexistence in these models is only possible under specific conditions, such as when mutualistic benefits saturate at high densities or are offset by strong self‐limitation (e.g. density dependence; Holland *et al*., 2002). Despite these challenges in modelling, recent advances by Hart (2023) are integrating facilitative interactions into classical models, utilising coexistence theory to refine our understanding of mutualistic and commensal relationships. Hart (2023) categorised facilitation within these models, highlighting a shift in coexistence theory from competition to facilitative interactions and stressing the importance of reciprocal interactions and density dependence in ecological outcomes. Integrating empirical and theoretical work is essential for a deeper understanding of the role of facilitation in population to ecosystem dynamics, and for predicting responses to future changes.

## V. IMPLICATIONS OF FACILITATIVE INTERSPECIES INTERACTIONS FOR COMMUNITIES AND ECOSYSTEMS

Compelling evidence indicates that facilitators – often mediated by foundation species, keystone structures, and ecosystem engineers (see Table 1) – can trigger strong direct and indirect effects that cascade through ecological communities, shaping both structure and function (Paine, 1966; Thomsen *et al*., 2010; Thomsen & Wernberg, 2014; Stachowicz, 2001; Witman, 1987; Stachowicz & Hay, 1999; Lafferty & Suchanek, 2016). Within the framework proposed by Thomsen *et al*. (2010), such interactions may propagate *via* “keystone” or “cascading” facilitation pathways. These facilitators often enhance biodiversity and ecological resilience, with keystone species contributing through biogenic habitat provision (Paine, 1966; Dayton, 1975; Jones, Lawton & Shachak, 1997; Lafferty & Suchanek, 2016), and Stachowicz (2001) argued that they can promote diversity similarly to predation by suppressing dominant competitors. The primary mechanism involves habitat‐forming organisms functioning as ecosystem engineers (Jones *et al*., 1994); by modifying the physical environment – altering sedimentation, nutrient dynamics, water flow, or structural complexity – they provide refuge, enhance local productivity, reduce environmental stress, and mitigate predation risks, thus facilitating other species' survival, growth, or reproduction (Bertness & Leonard, 1997; Bulleri, 2009; Bertness & Callaway, 1994; Jones *et al*., 1994). However, most of this empirical research has focused on sessile or sedentary groups, such as mussel beds, oyster reefs, coral reefs, macroalgae canopies, seagrasses, salt marshes, and mangroves (Stachowicz, 2001; Bulleri, 2009), whereas mobile marine vertebrates remain comparatively understudied as facilitators.

While direct behavioural interactions are the focus of this review, it is important first to recognise that mobile vertebrates also act as powerful facilitators through indirect, ecosystem‐engineering pathways. For instance, the “whale pump” – nutrient‐rich faeces released by whales near the surface – enhances primary productivity, increasing phytoplankton and potentially supporting larger primary consumer populations (Roman & McCarthy, 2010). Similarly, seabird guano provides substantial nutrient inputs to coastal ecosystems, fertilising primary producers and influencing the abundance and reproductive success of organisms across trophic levels (Polis, Anderson & Holt, 1997). These bottom‐up, nutrient‐mediated pathways demonstrate how ecosystem‐level facilitation directly shapes the productivity and persistence of recipient populations.

Beyond these indirect engineering roles, mobile marine vertebrates are also central to community dynamics through the direct behavioural interactions that are the primary focus of this review. For instance, Goyert *et al*. (2014) linked common tern (*Sterna hirundo*) and roseate tern (*Sterna dougallii*) distribution and abundance to associations with subsurface predators (tunas and dolphins) in the North Atlantic, exploring the implications of commensalism and local enhancement at the community level. This research classified tunas as “keystone facilitators” due to their impact on forage fish (Ashmole & Ashmole, 1967; Au & Pitman, 1986), thus impacting tern assemblages. Such interactions demonstrate how prey abundance dictates whether species compete under low prey availability or engage in mutualistic/commensal relationships to facilitate predation (Goyert *et al*., 2014; Au & Pitman, 1986). Under scarcity, terns may experience increased competition, leading to agonistic interactions or spatial segregation. Conversely, abundance or concentration of prey by facilitators like tunas allows species to benefit from information transfer and local enhancement, where individuals use others' presence and foraging success to locate and exploit prey more efficiently. This dynamic interplay highlights the context‐dependent nature of species interactions along a competition–facilitation spectrum.

This context‐dependent dynamic extends beyond the nature of the interaction to the very composition of the interacting groups themselves. Gostischa, Massolo & Constantine (2021) extensively explored mixed‐species feeding associations (MSFAs) among large marine predators (seabirds, cetaceans, sharks, and seals) analysing association patterns based on species composition, temporality, and prey preferences. They identified three MSFA categories by feeding habits and functional group composition. Over a decade, MSFA composition and dynamics showed temporal variability, emphasising the sensitivity of these facilitative communities to long‐term environmental changes. Such findings underscore the need to understand that the structure of these communities is not static, but shifts over time, which in turn influences the costs and benefits for all participants.

These shifting costs and benefits are often determined by the spatial co‐occurrence and composition of the groups. Indeed, studies on dynamic MSGs (seabirds, cetaceans, fish, and pinnipeds) emphasise how the net benefit for an individual fluctuates with group composition (Ashmole & Ashmole, 1967; Anderwald *et al*., 2011; Au & Pitman, 1986; Hebshi *et al*., 2008). A clear example is the commensal relationship observed in Norwegian fjords, where humpback whales (*Megaptera novaeangliae*) benefit from the presence of killer whales (*Orcinus orca*) while foraging on herring (*Clupea harengus*) (Jourdain & Vongraven, 2017). Although some group members may experience temporary disadvantages, these associations persist, demonstrating the resilience and adaptability of marine communities. This variation in group composition and community dynamics is often driven by environmental factors, as illustrated by Correia *et al*.'s (2019) investigation of MSGs of terns and subsurface predators during the terns' non‐breeding period in West Africa's shallow marine waters. They observed various interspecific interactions among tern species, with black terns (*Chlidonias niger*) foraging with heterospecifics in 89% of observations, indicating high dependence. The study highlighted how MSG composition varied with water depth: smaller predators like jacks (*Caranx hippos*) in shallow waters and larger predators like tunas, cetaceans, and pinnipeds in deeper waters. These insights reveal the complex dynamics of interspecific interactions, emphasising how environmental factors like water depth shape group composition.

Shifting the focus beyond apex predators, recent research on fish has explored community‐ and ecosystem‐level facilitation using interaction webs and network theory, particularly in coral reef ecosystems. For example, Fernández‐Cisternas *et al*. (2021) examined empirical facilitative interactions – such as mutualistic cleaning and following behaviour – within reef fish assemblages around Rapa Nui and Robinson Crusoe Islands. Their analysis also incorporated trophic interactions enabling indirect facilitation, such as predator‐mediated effects and trophic cascades. The study revealed how both top‐down pressures (e.g. fisheries) and bottom‐up drivers (e.g. productivity) shape the structure and resilience of facilitative networks. Robinson Crusoe Island exhibited a robust network, with a balance of agonistic and facilitative links contributing to higher community stability, whereas Rapa Nui's more fragile network suggested greater vulnerability to perturbations.

While such empirical studies advance our understanding of facilitation in marine networks, many interaction web models (e.g. Kéfi *et al*., 2015, 2016) are largely based on inferred or assumed interactions. These conceptual models are useful for exploring potential ecological outcomes, but they require validation with field data to assess the strength and direction of actual species interactions. Moreover, indirect facilitation is often already embedded in classical food‐web structures, where trophic pathways link species in complex but ecologically meaningful ways (e.g. species A suppresses predator B of species C). Mutualistic networks have been extensively studied in terrestrial ecosystems (e.g. plant–pollinator systems) and, to a lesser extent, in marine contexts such as anemone–fish associations (but see marine cleaning mutualisms, e.g. Quimbayo *et al*., 2018). An alternative empirical approach involves individual‐based network analyses that document species co‐occurrence and spatial associations without presupposing inhibition or facilitation (Thomsen & South, 2019). These approaches are promising for uncovering patterns of facilitation in dynamic, multi‐species marine systems.

Providing such an empirical approach, Auster *et al*. (2019) analysed a piscivorous fish assemblage engaged in group hunting within the coral reef ecosystem of Isla del Coco, Costa Rica. Using a network approach to study collective behaviour, they identified dominant species and highlighted the importance of quantifying facilitative interaction attributes within trophic guilds. This research sheds light on cooperative behaviours underpinning group hunting dynamics and contributes to conservation strategies by emphasising facilitative roles essential to ecosystem integrity.

Taken together, these examples illustrate that FII influences marine ecosystems *via* direct and indirect pathways. By integrating concepts such as interaction networks, keystone facilitation, niche modification, and non‐trophic mutualisms, we emphasise that facilitation is not limited to pairwise benefits but can restructure entire communities. Viewing facilitation through these ecological lenses reveals it as a powerful yet underappreciated mechanism shaping biodiversity, resilience, and trophic dynamics in marine systems.

## VI. INTEGRATING FACILITATIVE INTERACTIONS IN ECOSYSTEM‐BASED MANAGEMENT

As discussed above, FIIs play a critical role in marine ecosystems, influencing individual fitness, population dynamics, and community structure. These positive interactions, involving both apex predators and lower trophic‐level species, can shape food‐web resilience and ecosystem function. Yet, despite their ecological significance, FII remains largely absent from ecosystem models used in EBFM. EBFM frameworks seek to integrate ecological, social, and economic dimensions to manage fisheries sustainably (Link, 2010). To support this goal, a variety of ecosystem models (EMs) have been developed that differ in structure, assumptions, and complexity (Plagányi, 2007; Table 2). These models provide insight into the cumulative effects of fishing and environmental change by treating ecosystems as dynamic, interconnected systems. However, most EMs emphasise antagonistic interactions – such as predation and competition – while positive interspecific relationships are rarely represented. This limits their ability to capture the full complexity of marine ecosystem dynamics and may overlook important mechanisms that support biodiversity and ecosystem stability.

**Table 2:** Ecosystemic models that currently lack integration of positive interactions. Model classifications and abbreviations are based on Plagányi (2007), while ecosystem model (EM) references serve as primary data sources, complemented by papers illustrating model applications. Descriptions of facilitative interspecific interactions (FIIs) offer examples of such dynamics within specific regions or involving taxa similar to those discussed in the EM papers.

Model category	EM examples	EM reference	EM paper	FII description	FII reference
Mass‐balance	ECOPATH	www.ecopath.org	Olson & Watters (2003)	Tuna, dolphins, and seabirds foraging	Au & Pitman (1986)
Biogeochemical	ATLANTIS	https://research.csiro.au/atlantis/	Ainsworth *et al*. (2011)	Marine mammals foraging	Bacon *et al*. (2017)
Minimum Realistic	MSM	Jurado‐Molina *et al*. (2005)	Jurado‐Molina *et al*. (2005)	Gray whales and seabirds foraging	Grebmeier & Harrison (1992)
	MSVPA	Helgason & Gislason (1979) Pope (1979, 1991) Sparre (1993)	Pope & Macer (1991)	Seabirds foraging	Camphuysen *et al*. (2009)
	GADGET	www.hafro.is/gadget/	Trenkel *et al*. (2004)	Minke whales and seabirds foraging	Anderwald (2011)
Agent‐based	INVITRO	https://www.cmar.csiro.au/research/mse/invitro.htm	McDonald *et al*. (2008)	Cleaning mutualism	Caves (2021a)
Individual‐based	OSMOSE	https://osmose‐model.org	Fu *et al*. (2020)	Marine mammals and seabirds foraging	Pierotti (1988)
Size‐based	MIZER	https://sizespectrum.org/mizer/index.html	Woodworth‐Jefcoats *et al*. (2019)	Tuna, dolphins and seabirds foraging	Hebshi *et al*. (2008)
Bioenergetic	Yodzis & Innes (1992)	Yodzis & Innes (1992) Yodzis (1998) Koen‐Alonso & Yodzis (2005)	Koen‐Alonso & Yodzis (2005)	Pinnipeds and seabirds foraging	Thiebot & Weimerskirch (2013)

One of the most widely used tools for EBFM is Ecopath with Ecosim (EwE), a modelling suite that simulates energy flow and trophic dynamics in marine ecosystems. We discuss this model in more detail here to illustrate our argument regarding the inclusion of FII in models for EBFM. Initially developed by Polovina (1984) and expanded by Christensen & Pauly (1992), the Ecopath component constructs static, mass‐balance models of trophic interactions based on species biomass, diet composition, and fishing mortality. Its dynamic counterpart, Ecosim, incorporates temporal processes using differential equations to simulate population changes in response to fishing pressure, environmental variability, and habitat change (Walters, Christensen & Pauly, 1997; Ahrens, Walters & Christensen, 2012). Together, these tools enable scenario testing, long‐term trend analysis, and adaptive management planning (Christensen & Walters, 2004; Walters *et al*., 2005).

Despite EwE's broad application – evident in the 477 models archived in the EcoBase database (Colléter *et al*., 2015) – none currently incorporate FII involving apex predators or forage fish. This absence reflects the model's default structure, designed to track energy transfer through antagonistic links, illustrating a critical gap and a broader historical bias in ecological modelling towards competition and predation. This bias risks neglecting the stabilising or diversity‐enhancing effects of FII. However, EwE offers potential avenues for incorporating facilitation. The ‘mediation’ function can modify trophic interactions based on the biomass of a third species, offering a practical route for modelling facilitation. For example, a facilitating species could increase prey vulnerability or enhance foraging efficiency for another predator – mechanisms common in MSFAs.

Additionally, the vulnerability parameter in Ecosim, which controls the strength of top‐down *versus* bottom‐up control in predator–prey interactions, can be adapted to reflect facilitative mechanisms. By increasing prey vulnerability to a specific predator when facilitation occurs (e.g. through cooperative hunting), users can simulate enhanced foraging efficiency or prey accessibility in scenarios involving positive interspecific interactions. Adapting EwE and similar models to include facilitation would be a major step forward for EBFM. FIIs can modulate species coexistence, buffer ecosystems against perturbations, and influence community assembly – dynamics essential for anticipating ecological responses to fishing and climate change (see Section VII). Integrating these interactions would enhance the ecological realism of EMs, support better‐informed management strategies, and help safeguard marine biodiversity in an era of increasing human impact.

## VII. CONSERVATION RELEVANCE

Facilitative dynamics may profoundly impact species ecology (Bruno *et al*., 2003; Bronstein, 1994; Stachowicz, 2001), and here we examine their implications for conservation success and biodiversity. Conservation efforts prioritise protecting endangered, threatened, or vulnerable species and their habitats to prevent extinction and preserve biodiversity (Pullin, 2002; Lindenmayer & Burgman, 2005). Frameworks like ‘umbrella’ and ‘keystone’ species enhance conservation planning by highlighting species' ecological roles and significance within ecosystems (Pullin, 2002; Lindenmayer & Burgman, 2005; Caro, 2010; Barua, 2011). Umbrella species are typically large, wide‐ranging organisms whose conservation benefits numerous cohabiting species, while keystone species are vital to maintaining ecosystem structure and function (Pullin, 2002; Lindenmayer & Burgman, 2005; Caro, 2010; Barua, 2011). Recognising and protecting these species maximises conservation and biodiversity outcomes. Studies on FII have introduced the idea of ‘keystone facilitators’ (Au & Pitman, 1986; Ashmole & Ashmole, 1967; Stachowicz, 2001; Goyert, Manne & Veit, 2014; Goyert *et al*., 2018), which attract other species to MSGs through their behaviours (Goodale *et al*., 2017). Identifying such facilitators may further improve conservation strategies (Gostischa *et al*., 2021; Goyert *et al*., 2014; Veit & Harrison, 2017).

Moreover, keystone and umbrella species often serve as ‘flagship species’ due to their charisma, aesthetic appeal, or cultural significance (Caro, 2010; Barua, 2011). Understanding these species, particularly in the context of MSGs, strengthens conservation by utilising their emblematic status for broader actions. Many marine apex predators discussed herein (whales, dolphins, pinnipeds, albatrosses, and sharks) are flagship species (IUCN, 2024). These groups face significant threats from fisheries (Wade *et al*., 2021; Žydelis, Small & French, 2013; Pott & Wiedenfeld, 2017), with bycatch impacting various taxa (Dulvy *et al*., 2021) and overfishing affecting sharks and tunas (Hunter *et al*., 1986; Kompas, Grafton & Che, 2010). While some apex predators like sharks influence ecosystems through trophic cascades (e.g. mesopredator suppression), they are not necessarily facilitative as defined here, as they do not provide non‐trophic benefits to other species. Clarifying this distinction avoids conflating top‐down control with facilitation. Thus, conservation should also prioritise less‐charismatic but ecologically vital facilitative species that directly contribute to community structure and resilience.

The ecosystem‐based conservation approach is widely regarded as effective for preserving ecosystem structure and functionality (Garcia & Cochrane, 2005). Conservation biology emphasises identifying keystone, umbrella, and flagship species, which are central to this ecosystemic paradigm, as their protection is expected to create cascading benefits across species networks, including FIIs. Furthermore, this approach integrates socio‐economic considerations (Long, Charles & Stephenson, 2015; Curtin & Prellezo, 2010; Gruber, 2010; Garcia & Cochrane, 2005), promoting sustainable fisheries practices and stakeholder engagement *via* bioeconomic models (e.g. Azevedo *et al*., 2024). Employing ecosystem models that explicitly characterise facilitative interactions alongside antagonistic ones therefore will provide a more comprehensive strategy for species conservation.

**Fig. 3:**
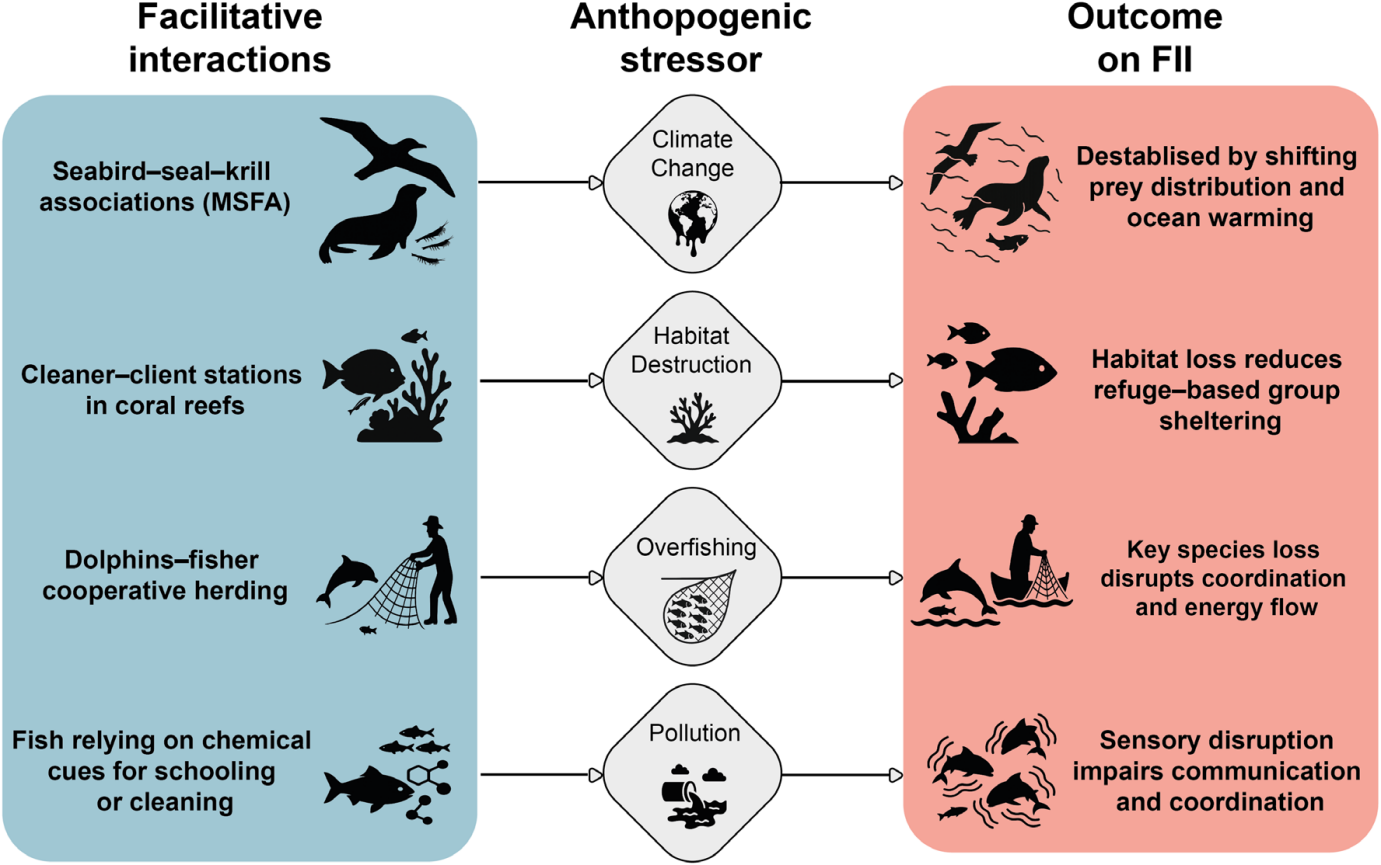
Schematic overview of how anthropogenic stressors impact facilitative interspecific interactions (FIIs) in marine ecosystems. The diagram presents four illustrative examples of FII (left column) and links them to major human‐driven stressors (centre column), showing their respective ecological consequences (right column). Arrows indicate the causal pathways through which stressors alter the structure, function, or persistence of these interactions. This conceptual synthesis highlights how different stressors can disrupt species coordination, communication, sheltering, and mutualistic behaviours essential to ecosystem functioning. MSFA, multi‐species feeding association.

Beyond their intrinsic ecological value, FIIs are increasingly shaped by anthropogenic pressures. The five major drivers of biodiversity loss – climate change, habitat destruction, resource extraction (e.g. overfishing), biological invasions, and pollution/eutrophication – can significantly disrupt FIIs across marine ecosystems. Among these, climate change has received the most attention. For example, shifts in ocean temperature and prey distribution have been shown to destabilise MSFAs involving krill predators such as seabirds and seals (Monier, Veit & Manne, 2020), while environmental variability can alter MSFA composition (Gostischa *et al*., 2021). By contrast, the effects of habitat degradation, pollution, overfishing, and invasive species on FIIs remain poorly understood. However, it is plausible that these stressors have significant impacts: habitat degradation could reduce refuge‐based facilitation; pollution may impair the sensory mechanisms essential for interspecific coordination; overfishing can remove key facilitator species; and invasive species may dismantle native facilitative networks. Despite these potential consequences (Fig. 3), FIIs are still largely overlooked in conservation planning. Explicitly incorporating FII into management frameworks could enhance ecosystem resilience and adaptive capacity in the face of global change.

## VIII. IMPLICATIONS FOR HUMAN–WILDLIFE INTERACTIONS

FIIs are also evident in human–wildlife contexts (van der Wal *et al*., 2022). A notable example occurs in southern Brazil, where artisanal fishers collaborate with wild bottlenose dolphins (*T. truncatus gephyreus*) to catch migratory mullet (*Mugil* spp.) (Simões‐Lopes, 1998; Cantor *et al*., 2023). In this traditional fishery, dolphins herd mullet towards shallow waters and produce cues that signal when and where fishers should cast their nets. Disoriented by the nets, mullet become easier prey, allowing dolphins to forage more effectively using echolocation (Cantor *et al*., 2023). This interaction is one of the few remaining examples of human–wildlife cooperation and represents a flagship ecological interaction, a rare but culturally and ecologically significant relationship that fosters conservation awareness and strengthens human–nature connections (see Table 1). When foraging in synchrony, dolphins are nearly three times more likely to succeed in hunting, while fishers are 17 times more likely to catch mullet (Cantor *et al*., 2023). This cooperation can scale up to influence population dynamics, community structure, and ecosystem‐level processes (Bezamat *et al*., 2018; Dalpaz *et al*., 2024; Wooster *et al*., 2023). However, this mutualism may shift towards commensalism or even kleptoparasitism depending on ecological conditions, particularly when synchrony is low or prey is scarce.

Beyond enhanced foraging, human–wildlife FIIs also generate non‐material benefits. For southern Brazilian fishers, these include cultural services such as a sense of place, belonging, and cooperation among fishers (Machado *et al*., 2019; Santos‐Silva *et al*., 2022). For dolphins, cooperation is associated with increased survival (Bezamat *et al*., 2018, 2021) and stronger social bonds (Daura‐Jorge *et al*., 2012; Daura‐Jorge, Ingram & Simões‐Lopes, 2013; Machado *et al*., 2019). Still, the interaction faces multiple threats, including changing and declining fisher participation (Smith *et al*., 2009; Cantor *et al*., 2024), overfishing (Santos, Lemos & Viera, 2018; Simões‐Lopes, 1998), and bycatch from illegal fishing (Bezamat *et al*., 2021; Peterson, Hanazaki & Simões‐Lopes, 2008). Recognising the importance of such FII in conservation planning is essential for ecosystem health, the maintenance of ecosystem services, and the well‐being of coastal communities.

## IX. FILLING THE GAPS AND FUTURE DIRECTIONS

### (1) Individual level

While FIIs have moved beyond being a niche topic in ecology (Carlson *et al*., 2023), significant knowledge gaps remain. Key questions focus on the behavioural differences between conspecifics engaged in FIIs and those typically involved in single‐species interactions. Establishing a framework to measure foraging efficiencies explicitly – such as bite rates, foraging duration, prey quality, and other metrics – between mixed‐ and single‐species groups could provide valuable insights into the comparative advantages and trade‐offs of these interaction types. Further research should also examine the level of behavioural coordination among parties in FIIs, aggregations, MSGs, or MSFAs. Additionally, investigating the necessity of communication for achieving common goals, as well as potential differences in communication strategies between conspecifics and heterospecifics, deserves further attention. Understanding how individuals balance trade‐offs between positive and negative outcomes is critical, as variations within populations ultimately shape the dynamics of interspecific interactions.

Farine, Garroway & Sheldon (2012) advocate for a ‘bottom‐up’ approach, focusing on individuals as the primary unit of analysis to deepen our understanding of FII dynamics. Social network analysis, grounded in individual‐centric rather than species‐centric paradigms, is pivotal to advancing this bottom‐up approach. Initially applied in avian studies, no theoretical barriers (other than logistical challenges in aquatic sampling) prevent extending individual‐based social network analysis to marine fish and apex predators, as explored by Krause, Croft & James (2007) and further analysed by Krause *et al*. (2015) and Croft, James & Krause (2008). Recent developments now enable statistical inference at the individual level. For example, Thomsen & South (2019) introduced frameworks allowing error estimation on individual nodes within ecological networks, offering more robust assessments of species interactions and their variability. However, understanding how the environment influences individual decisions and behaviours during mixed‐species encounters and sequential facilitation is a major challenge. Many FIIs occur in specific spatiotemporal contexts, underscoring the need to explore the interaction between environmental factors and individual behavioural responses. Such studies promise to illuminate the dynamics and ecological implications of FIIs while assessing the broader ecosystem effects of individual choices and behaviours.

### (2) Population level

As noted by Hart (2023), unlike research on competition and predation, studies on facilitation – aside from mutualism – lack a solid foundation in population dynamic theory. While mutualisms have been more extensively studied, other forms of facilitation remain less explored. Consequently, there has been less focus on estimating state variables and population‐level parameters that predict the outcomes of facilitative interactions. Despite these challenges, research has increasingly explored how facilitation influences population abundance, distribution, and demography (Bruno *et al*., 2003; Bulleri, 2009).

Several challenges persist in understanding the connections between FII and population ecology. As Agrawal *et al*. (2007) and Chu *et al*. (2008) have emphasised, population density and environmental factors determine whether interactions are mainly positive (facilitative) or negative (competitive). Evaluating how fish and marine apex predator population densities involved in FII influence population dynamics could yield valuable insights, particularly given environmental change. Cameron, Coulson & Marshall (2019), Lin *et al*. (2012, 2016), Malanson & Resler (2016), and Zepeda & Martorell (2019) have noted that both population density and individual size structure significantly impact the frequency and intensity of facilitation *versus* competition, affecting the life‐history traits of species involved.

Fish and marine apex predators could serve as focal groups to test the abiotic stress gradient hypothesis proposed by Bertness & Callaway (1994), which suggests that facilitation is more prevalent under high abiotic stress, while competition dominates under less‐stressful conditions. However, recent findings suggest strong facilitation can occur under low‐stress conditions, particularly when interactions are driven by predator avoidance rather than environmental harshness (Kawai & Tokeshi, 2007). For example, small fish shelter among jellyfish tentacles in benign pelagic environments, gaining protection from predators (Griffin *et al*., 2019). This suggests a unimodal pattern between stress and facilitation, peaking at moderate stress levels and declining at both extremes (Michalet *et al*., 2006, 2014; Xiao *et al*., 2009; Holmgren & Scheffer, 2010; Verwijmeren *et al*., 2013; Le Bagousse‐Pinguet *et al*., 2014). Testing this hypothesis in diverse ecological contexts, particularly for mobile fauna, could clarify whether FIIs involving fish and marine apex predators also intensify under high environmental stress, as the theory was initially developed for foundation species that facilitate through physical habitat modification, like mussel beds, coral reefs, and mangroves.

Additionally, examining the demographic aspects of fish and apex predator populations involved in FII could deepen our understanding of population dynamics, as suggested by Caswell (2006). Demographic models provide a framework for integrating empirically estimated rates, such as survival, growth, and reproduction, across life stages to project future population growth under current ecological conditions. Analysing species distribution ranges could also shed light on how facilitation influences population dynamics, as highlighted in studies by Bulleri *et al*. (2016) and Butterfield (2009).

### (3) Community and ecosystem levels

McIntire & Fajardo (2014) highlight facilitation as a key driver of biodiversity, as species modify local biotic or abiotic conditions through resource exploitation, thus reshaping multidimensional niches. This alteration can shift species composition, often enhancing both beta and, occasionally, alpha diversity. The importance of facilitation is amplified in complex communities where species not only exploit but also create niches *via* interactions with the environment. While McIntire & Fajardo's (2014) work focused primarily on sessile organisms, all organisms, regardless of mobility, can influence their environment and shape ecological outcomes. Foundational studies on facilitation by sessile or low‐mobility organisms (e.g. Bertness & Callaway, 1994; Dayton, 1972; Jones *et al*., 1994, 1997; Ellison *et al*., 2005; Tews *et al*., 2004; Townsend, 2021) have underscored the importance of foundation species, keystone structures, and allogenic ecosystem engineers in promoting biodiversity and stabilising communities.

However, recent research shows that facilitation also plays a critical role among mobile organisms. Although historically overlooked, mobile species can function as biodiversity reservoirs, expanding the conceptual scope of facilitation (Altieri & Witman, 2014). Despite logistical challenges such as spatial and temporal segregation (e.g. Karban, Grof‐Tisza & Holyoak, 2012; Li *et al*., 2018), facilitative interactions among mobile species are increasingly recognised as ecologically relevant, contributing to coexistence, community resilience, and ecosystem functioning (e.g. Canepuccia *et al*., 2020; van der Wal *et al*., 2022). Such interactions often involve one species enhancing another's access to or quality of resources, influencing species distributions and community dynamics, potentially driving biodiversity (e.g. Bronstein, 2009; Hale, Valdovinos & Martinez, 2020).

Canepuccia *et al*. (2024) demonstrate how indirect and reciprocal facilitative effects intricately influence species' behaviour, abundance, and resource use within interaction networks. These modifications apply selection pressures across communities, expanding niches and enhancing biodiversity. Empirical evidence across botanical contexts consistently underscores the importance of facilitation for ecosystem structure, function, and dynamics (Callaway, 2007). Understanding facilitation mechanisms and their broader ecosystem impacts is critical, especially for mobile species like fish and apex predators. Michalet & Pugnaire (2016), for instance, suggest that understanding the role of facilitation could improve ecosystem management and restoration by leveraging positive feedback loops to avert catastrophic shifts. Fully comprehending species‐level facilitation and its ecosystem‐level implications requires investigating complete interaction networks among species and their abiotic environments, merging ecosystem and community ecology approaches (Kéfi *et al*., 2016).

Ecosystem modelling has explored facilitation in diverse contexts, although gaps remain in addressing interactions among mobile species. Ecopath models have considered facilitation, such as those of Harvey (2014), who explored eelgrass (*Zostera marina*) as a refuge habitat for juvenile Pacific salmon (*Oncorhynchus* spp.). However, scaling mediation functions to ecosystem levels poses challenges in Ecopath, as detailed by Espinosa‐Romero *et al*. (2011) and Plummer *et al*. (2013). Harvey (2014) emphasises the need for exploratory approaches to refine model assumptions, while Espinosa‐Romero *et al*. (2011) exemplify the application of Ecopath's mediation function, showing how kelps enhance feeding areas and food availability for other species, significantly affecting biomass and energy flow of fish, benthic invertebrates, otters, and detritus. Although these findings diverge from this review's focal groups, they underscore the potential value of integrating facilitation within an ecosystem framework. In addition to the mediation function, the Ecosim's ‘vulnerability’ parameter also provides a promising tool to represent facilitation, allowing predator–prey interactions to shift based on the presence or behaviour of a facilitator, consistent with the principles of the foraging arena theory (Walters *et al*., 1997).

## X. CONCLUSIONS

(1) This review synthesises the multifaceted roles of facilitative interspecific interactions (FIIs) across multiple biological scales in the marine environment, connecting individual‐level behaviours to their broader consequences for populations, communities, and ecosystems.
(2) Significant progress has been made in descriptive studies over recent decades; however, important gaps remain in understanding the impacts of FIIs on population dynamics, community structure, and ecosystem processes within marine environments.
(3) Facilitation has been widely studied in sessile species, often supported by experimental evidence and long‐term demographic data demonstrating clear positive effects on population dynamics. By contrast, facilitation among pelagic animals remains less experimentally tested, with most studies relying on correlations. While these correlations suggest potential positive interactions, the absence of long‐term demographic data makes it difficult to confirm facilitation in the strictest sense. Future studies incorporating experimental manipulations or long‐term monitoring could help establish causal relationships and clarify the demographic consequences of these interactions.
(4) Our synthesis also highlights a key research gap: despite growing recognition of the importance of FIIs, few studies have quantified their outcomes in terms of reproductive success, population growth, or community stability. Addressing this gap will be essential for improving ecological models, informing conservation strategies, and distinguishing facilitation more clearly from other ecological processes such as trophic interactions.
(5) We strongly recommend incorporating FII into ecosystem models to improve Ecosystem‐Based Fishery Management (EBFM). This is particularly critical for conserving vulnerable taxa, including fish, sharks, seabirds, cetaceans, and pinnipeds, whose populations may depend on these essential interactions.
(6) Finally, ensuring the resilience of human communities that rely on ocean ecosystems is an urgent priority. In this context, FIIs involving humans and apex predators pose unique management challenges, yet they also offer important opportunities for expanded data collection, model development, and understanding broader ecological and socio‐economic impacts.

## AUTHOR CONTRIBUTIONS

E. D. V.: Conceptualisation; Data collection; Data curation; Investigation; Methodology; Visualisation; Writing – original draft. F. G. D‐J.: Conceptualisation; Data collection; Formal analysis; Methodology; Funding acquisition; Project administration; Writing – original draft; review & editing; Supervision; Validation; Visualisation; J. L. R‐F.: Investigation; Resources; Supervision; Writing – review & editing; R. A.: Investigation; Resources; Supervision; Writing – review & editing; M. C.: Investigation; Resources; Writing – review & editing; S. S.: Conceptualisation; Data collection; Formal analysis; Methodology; Writing – original draft; review & editing; Supervision;

## References

[brv70091-bib-0001] Acevedo‐Gutiérrez, A. (1999). Aerial behavior is not a social facilitator in bottlenose dolphins hunting in small groups. Journal of Mammalogy 80(3), 768–776.

[brv70091-bib-0002] Agrawal, A. A. , Ackerly, D. D. , Adler, F. , Arnold, A. E. , Cáceres, C. , Doak, D. F. , Post, E. , Hudson, P. J. , Maron, J. L. , Mooney, K. A. , Power, M. , Schemske, D. , Stachowicz, J. , Strauss, S. , Turner, M. G. & Werner, E. (2007). Filling key gaps in population and community ecology. Frontiers in Ecology and the Environment 5(3), 145–152.

[brv70091-bib-0003] Ahrens, R. N. , Walters, C. J. & Christensen, V. (2012). Ecosim: modelling ecosystem dynamics over time. Fisheries Centre Research Reports 20(2), 1–99.

[brv70091-bib-0004] Ainsworth, C. H. , Samhouri, J. F. , Busch, D. S. , Cheung, W. W. , Dunne, J. & Okey, T. A. (2011). Potential impacts of climate change on Northeast Pacific marine foodwebs and fisheries. ICES Journal of Marine Science 68(6), 1217–1229.

[brv70091-bib-0005] Altieri, A. H. & Witman, J. D. (2014). Modular mobile foundation species as reservoirs of biodiversity. Ecosphere 5(10), 1–11.

[brv70091-bib-0006] Anderwald, P. (2011). *Ecology and behaviour of minke whales in the Northeast Atlantic*. Doctoral Dissertation. University of Aberdeen.

[brv70091-bib-0007] Anderwald, P. , Evans, P. G. , Gygax, L. & Hoelzel, A. R. (2011). Role of feeding strategies in seabird‐minke whale associations. Marine Ecology Progress Series 424, 219–227.

[brv70091-bib-0008] Ashmole, N. P. & Ashmole, M. J. (1967). Comparative feeding ecology of sea birds of a tropical oceanic Island. Bulletin of the Peabody Museum of Natural History 40(1), 1–131.

[brv70091-bib-0009] Au, D. W. (1991). Polyspecific nature of tuna schools: shark, dolphin, and seabird associates. Fishery Bulletin 89(3), 343–354.

[brv70091-bib-0010] Au, D. W. K. & Pitman, R. L. (1986). Seabird interactions with dolphins and tuna in the eastern tropical Pacific. The Condor: Ornithological Applications 88(3), 304–317.

[brv70091-bib-0011] Au, D. W. & Pitman, R. L. (1988). Seabird relationships with tropical tunas and dolphins. In Seabirds and Other Marine Vertebrates, pp. 174–212. Columbia University Press, New York.

[brv70091-bib-0012] Auster, P. J. , Cortés, J. , Alvarado, J. J. & Beita‐Jiménez, A. (2019). Coordinated hunting behaviors of mixed‐species groups of piscivores and associated species at Isla del coco National Park (eastern tropical Pacific). Neotropical Ichthyology 17(1), 1–12.

[brv70091-bib-0013] Azevedo, E. , Pintassilgo, P. , Dantas, D. & Daura‐Jorge, F. G. (2024). A bioeconomic model for a multispecies small‐scale fishery system. Ecological Economics 226, 108358.

[brv70091-bib-0014] Bacon, J. P. , Reid, R. N. & Livingston, P. A. (2017). Marine mammals as ecosystem sentinels: foraging impacts in the Bering Sea. Marine Ecology Progress Series 573, 187–202.

[brv70091-bib-0015] Bailey, I. , Myatt, J. P. & Wilson, A. M. (2013). Group hunting within the carnivora: physiological, cognitive and environmental influences on strategy and cooperation. Behavioral Ecology and Sociobiology 67(1), 1–17.

[brv70091-bib-0016] Baird, T. A. (1993). A new heterospecific foraging association between the puddingwife wrasse, *Halichoeres radiatus*, and the bar jack, *Caranx ruber*: evaluation of the foraging consequences. Environmental Biology of Fishes 38(4), 393–397.

[brv70091-bib-0017] Ballance, L. T. , Pitman, R. L. & Fiedler, P. C. (2006). Oceanographic influences on seabirds and cetaceans of the eastern tropical Pacific: a review. Progress in Oceanography 69(2–4), 360–390.

[brv70091-bib-0018] Baraza, E. , Zamora, R. , Hódar, J. A. & Gómez, J. M. (2007). Plant–herbivore interaction: beyond a binary vision. In Functional Plant Ecology. Second edition (eds F. I. Pugnaire and F. Valladares ), pp. 678–709. CRC Press, Boca Raton.

[brv70091-bib-0019] Barlow, G. W. (1972). A paternal role for bulls of the Galapagos Islands sea lion. Evolution 26(2), 307–310. 28555740 10.1111/j.1558-5646.1972.tb00196.x

[brv70091-bib-0020] Barua, M. (2011). Mobilizing metaphors: the popular use of keystone, flagship and umbrella species concepts. Biodiversity and Conservation 20, 1427–1440.

[brv70091-bib-0021] Bearzi, M. (2006). California sea lions use dolphins to locate food. Journal of Mammalogy 87(3), 606–617.

[brv70091-bib-0022] Beebe, W. (1928). Beneath Tropic Seas: A Record of Diving Among the Coral Reefs of Haiti. GP Putnam's Sons, New York & London.

[brv70091-bib-0023] Beltran, R. S. , Kilpatrick, A. M. , Picardi, S. , Abrahms, B. , Barrile, G. M. , Oestreich, W. K. , Smith, J. A. , Czapanskiy, M. F. , Favilla, A. B. , Reisinger, R. R. , Kendall‐Bar, J. M. , Payne, A. R. , Savoca, M. S. , Palance, D. G. , Andrzejaczek, S. , *et al*. (2024). Maximizing biological insights from instruments attached to animals. Trends in Ecology & Evolution 40(1), 37–46. 39472251 10.1016/j.tree.2024.09.009

[brv70091-bib-0024] Bertness, M. D. & Callaway, R. (1994). Positive interactions in communities. Trends in Ecology & Evolution 9(5), 191–193. 21236818 10.1016/0169-5347(94)90088-4

[brv70091-bib-0025] Bertness, M. D. & Leonard, G. H. (1997). The role of positive interactions in communities: lessons from intertidal habitats. Ecology 78(7), 1976–1989.

[brv70091-bib-0026] Bezamat, C. , Hammond, P. S. , Castilho, P. V. , Simões‐Lopes, P. C. & Daura‐Jorge, F. G. (2021). Dolphin population specialized in foraging with artisanal fishers requires zero‐bycatch management to persist. Aquatic Conservation: Marine and Freshwater Ecosystems 31(11), 3133–3145.

[brv70091-bib-0027] Bezamat, C. , Simões‐Lopes, P. C. , Castilho, P. V. & Daura‐Jorge, F. G. (2018). The influence of cooperative foraging with fishermen on the dynamics of a bottlenose dolphin population. Marine Mammal Science 35(3), 825–842.

[brv70091-bib-0028] Boucher, D. H. , James, S. & Keeler, K. H. (1982). The ecology of mutualism. Annual Review of Ecology and Systematics 13, 315–347.

[brv70091-bib-0029] Brewer, M. L. & Hertel, F. (2007). Wing morphology and flight behavior of pelecaniform seabirds. Journal of Morphology 268(10), 866–877. 17638303 10.1002/jmor.10555

[brv70091-bib-0030] Bronstein, J. L. (1994). Our current understanding of mutualism. The Quarterly Review of Biology 69(1), 31–51.

[brv70091-bib-0031] Bronstein, J. L. (2009). The evolution of facilitation and mutualism. Journal of Ecology 97(6), 1160–1170.

[brv70091-bib-0032] Bronstein, J. L. (ed.) (2015). Mutualism. Oxford University Press, Oxford.

[brv70091-bib-0033] Bronstein, J. L. & Sridhar, H. (2024). Connecting and integrating cooperation within and between species. Philosophical Transactions of the Royal Society of London Series B 379(1909), 20230203. 39034697 10.1098/rstb.2023.0203 PMC11293865

[brv70091-bib-0034] Bruno, J. F. , Stachowicz, J. J. & Bertness, M. D. (2003). Inclusion of facilitation into ecological theory. Trends in Ecology and Evolution 18, 119–125.

[brv70091-bib-0035] Bshary, R. (2003). The cleaner wrasse, *Labroides dimidiatus*, is a key organism for reef fish diversity at Ras Mohammed National Park, Egypt. Journal of Animal Ecology 72(1), 169–176.

[brv70091-bib-0036] Bshary, R. , Hohner, A. , Ait‐el Djoudi, K. & Fricke, H. (2006). Interspecific communicative and coordinated hunting between groupers and giant moray eels in the Red Sea. PLoS Biology 4(12), e431. 17147471 10.1371/journal.pbio.0040431 PMC1750927

[brv70091-bib-0037] Buchanan‐Smith, H. M. (1999). Tamarin polyspecific associations: Forest utilization and stability of mixed‐species groups. Primates 40, 233–247. 23179543 10.1007/BF02557713

[brv70091-bib-0038] Buckley, N. J. (1996). Food finding and the influence of information, local enhancement, and communal roosting on foraging success of north American vultures. The Auk 113(2), 473–488.

[brv70091-bib-0039] Bulleri, F. (2009). Facilitation research in marine systems: state of the art, emerging patterns, and insights for future developments. Journal of Ecology 97(6), 1121–1130.

[brv70091-bib-0040] Bulleri, F. , Bruno, J. F. , Silliman, B. R. & Stachowicz, J. J. (2016). Facilitation and the niche: implications for coexistence, range shifts, and ecosystem functioning. Functional Ecology 30(1), 70–78.

[brv70091-bib-0041] Butterfield, B. J. (2009). Effects of facilitation on community stability and dynamics: synthesis and future directions. Journal of Ecology 97(6), 1192–1201.

[brv70091-bib-0042] Callaway, R. M. (2007). Positive Interactions and Interdependence in Plant Communities. Springer, Dordrecht.

[brv70091-bib-0043] Cameron, H. , Coulson, T. & Marshall, D. J. (2019). Size and density mediate transitions between competition and facilitation. Ecology Letters 22(11), 1879–1888. 31468661 10.1111/ele.13381

[brv70091-bib-0044] Camphuysen, C. J. , Ensor, K. , Huppop, O. , Leaper, G. , Offringa, H. & Tasker, M. L. (2009). Seabird foraging and predator–prey interactions. ICES Journal of Marine Science 63(7), 1111–1121.

[brv70091-bib-0045] Canepuccia, A. D. , Alemany, D. , Vidal, E. E. , Alvarez, M. F. & Iribarne, O. O. (2020). Temporal variation in positive and negative interactions between marsh herbivores mediated by changes in plant traits. Marine Ecology Progress Series 634, 89–97.

[brv70091-bib-0046] Canepuccia, A. D. , Hidalgo, F. J. , Fanjul, E. & Iribarne, O. O. (2024). Reciprocal facilitation between ants and small mammals in tidal marshes. Oecologia 204(3), 575–588. 38376632 10.1007/s00442-024-05513-2

[brv70091-bib-0047] Cantor, M. , Farine, D. R. & Daura‐Jorge, F. G. (2023). Foraging synchrony drives resilience in human‐dolphin mutualism. Proceedings of the National Academy of Sciences of the United States of America 120(6), e2207739120. 36716378 10.1073/pnas.2207739120 PMC9963516

[brv70091-bib-0048] Cantor, M. , Santos‐Silva, B. , Daura‐Jorge, F. G. , Machado, A. M. S. , Peterson, D. , da‐Rosa, D. X. , Simões‐Lopes, P. C. , Valle‐Pereira, J. V. S. , Zank, S. & Hanazaki, N. (2024). Changes in the users of the social‐ecological system around a reciprocal human‐dolphin relationship. People and Nature 7, 974–989.

[brv70091-bib-0049] Carlson, N. V. , Freeberg, T. M. , Goodale, E. & Theo, A. H. (2023). Mixed‐species groups and aggregations: shaping ecological and behavioural patterns and processes. Philosophical Transactions of the Royal Society B: Biological Sciences 378(1878), 20220093. 10.1098/rstb.2022.0093 PMC10107224 37066660

[brv70091-bib-0050] Caro, T. M. (2010). Conservation by Proxy: Indicator, Umbrella, Keystone, Flagship, and Other Surrogate Species. Island Press, Washington, DC.

[brv70091-bib-0051] Caswell, H. (2006). Evolutionary demography: the invasion exponent and the effective population density in nonlinear matrix models. In From Energetics to Ecosystems: The Dynamics and Structure of Ecological Systems (Volume 1, eds N. Rooney , K. S. McCann , and D. L. G. Noakes ), pp. 237–256. Springer, Dordrecht.

[brv70091-bib-0052] Caves, E. M. (2021 *a*). Cleaning mutualisms in marine systems: a review. Annual Review of Marine Science 13, 205–227.

[brv70091-bib-0053] Caves, E. M. (2021 *b*). The behavioural ecology of marine cleaning mutualisms. Biological Reviews 96(6), 2584–2601. 34165230 10.1111/brv.12770

[brv70091-bib-0054] Ceyrac, L. , Barreau, E. , Modi, A. , Estrade, V. & Dulau, V. (2018). Using passive acoustic monitoring to assess humpback whale occurrence and breeding activity around La Réunion Island. Western Indian Ocean Journal of Marine Science Special Issue 1, 65–73.

[brv70091-bib-0055] Chase, I. D. (1980). Cooperative and noncooperative behavior in animals. The American Naturalist 115(6), 827–857.

[brv70091-bib-0056] Chesson, P. (2000). Mechanisms of maintenance of species diversity. Annual Review of Ecology and Systematics 31, 343–366.

[brv70091-bib-0057] Christensen, V. & Pauly, D. (1992). Ecopath ii–a software for balancing steady‐state ecosystem models and calculating network characteristics. Ecological Modelling 61(3–4), 169–185.

[brv70091-bib-0058] Christensen, V. & Walters, C. J. (2004). Ecopath with ecosim: methods, capabilities, and limitations. Ecological Modelling 172(2–4), 109–139.

[brv70091-bib-0059] Chu, C. J. , Maestre, F. T. , Xiao, S. , Weiner, J. , Wang, Y. S. & Duan, Z. H. (2008). Balance between facilitation and resource competition determines biomass–density relationships in plant populations. Ecology Letters 11(11), 1189–1197. 18684118 10.1111/j.1461-0248.2008.01228.x

[brv70091-bib-0060] Clayton, H. , Cade, D. E. , Burnham, R. , Calambokidis, J. & Goldbogen, J. (2023). Acoustic behavior of gray whales tagged with biologging devices on foraging grounds. Frontiers in Marine Science 10, 1111666.

[brv70091-bib-0061] Clua, É. & Grosvalet, F. (2001). Mixed‐species feeding aggregation of dolphins, large tunas and seabirds in the Azores. Aquatic Living Resources 14(1), 11–18.

[brv70091-bib-0062] Clutton‐Brock, T. (2009). Cooperation between non‐kin in animal societies. Nature 462(7269), 51–57. 19890322 10.1038/nature08366

[brv70091-bib-0063] Colléter, M. , Valls, A. , Guitton, J. , Gascuel, D. , Pauly, D. & Christensen, V. (2015). Global overview of the applications of the Ecopath with Ecosim modeling approach using the EcoBase models repository. Ecological Modelling 302, 42–53.

[brv70091-bib-0064] Connor, R. C. (2000). Group living in whales and dolphins. In Cetacean Societies: Field Studies of Dolphins and Whales (eds J. Mann , R. C. Connor , P. L. Tyack , and H. Whitehead ), pp. 199–218. University of Chicago Press, Chicago.

[brv70091-bib-0065] Correia, E. , Granadeiro, J. P. , Mata, V. A. , Regalla, A. & Catry, P. (2019). Trophic interactions between migratory seabirds, predatory fishes, and small pelagics in coastal West Africa. Marine Ecology Progress Series 622, 177–189.

[brv70091-bib-0066] Cram, D. L. , Van Der Wal, J. E. M. , Uomini, N. , Cantor, M. , Afan, A. I. , Attwood, M. C. , Amphaeris, J. , Balasani, F. , Blair, C. J. , Bronstein, J. L. , Buanachique, I. O. , Cuthill, R. R. T. , Das, J. , Daura‐Jorge, F. G. , Deb, A. , *et al*. (2022). The ecology and evolution of human‐wildlife cooperation. People and Nature 4(4), 841–855.

[brv70091-bib-0067] Croft, D. P. , James, R. & Krause, J. (2008). Exploring Animal Social Networks. Princeton University Press, Princeton.

[brv70091-bib-0068] Crowley, P. H. & Cox, J. J. (2011). Intraguild mutualism. Trends in Ecology & Evolution 26(12), 627–633. 21880393 10.1016/j.tree.2011.07.011

[brv70091-bib-0069] Curtin, R. & Prellezo, R. (2010). Understanding marine ecosystem‐based management: a literature review. Marine Policy 34(5), 821–830.

[brv70091-bib-0070] Dalpaz, L. , Daura‐Jorge, F. G. , Lewison, R. , Zank, S. & Hanazaki, N. (2024). Fishers' perception and activity shifts in a dolphin bycatch mitigation context. Ocean & Coastal Management 258, 107375.

[brv70091-bib-0071] Daura‐Jorge, F. G. , Cantor, M. , Ingram, S. N. , Lusseau, D. & Simões‐Lopes, P. C. (2012). The structure of a bottlenose dolphin society is coupled to a unique foraging cooperation with artisanal fishermen. Biology Letters 8(5), 702–705. 22552635 10.1098/rsbl.2012.0174 PMC3440962

[brv70091-bib-0072] Daura‐Jorge, F. G. , Ingram, S. N. & Simões‐Lopes, P. C. (2013). Seasonal abundance and adult survival of bottlenose dolphins (*Tursiops truncatus*) in a community that cooperatively forages with fishermen in southern Brazil. Marine Mammal Science 29(2), 293–311.

[brv70091-bib-0073] Dayton, P. K. (1972). Toward an understanding of community resilience and the potential effects of enrichments to the benthos at McMurdo Sound, Antarctica. Proceedings of the Colloquium on Conservation Problems in Antarctica, 81–96.

[brv70091-bib-0074] Dayton, P. K. (1975). Experimental evaluation of ecological dominance in a rocky intertidal algal community. Ecological Monographs 45(2), 137–159.

[brv70091-bib-0075] Dickie, J. (2005). Mobbing of a great white shark (*Carcharodon carcharias*) by adult male Australian fur seals (*Arctocephalus pusillus doriferus*). Marine Mammal Science 21, 336–339.

[brv70091-bib-0076] Dugatkin, L. A. (2002). Cooperation in animals: an evolutionary overview. Biology and Philosophy 17(4), 459–476.

[brv70091-bib-0077] Dulvy, N. K. , Pacoureau, N. , Rigby, C. L. , Pollom, R. A. , Jabado, R. W. , Ebert, D. A. , Finucci, B. , Pollock, C. M. , Cheok, J. , Derrick, D. H. , Herman, K. B. , Sherman, C. S. , Vanderwright, W. J. , Lawson, J. M. , Walls, R. H. L. , Carlson, J. K. , *et al*. (2021). Overfishing drives over one‐third of all sharks and rays toward a global extinction crisis. Current Biology 31(21), 4773–4787.e8. 34492229 10.1016/j.cub.2021.08.062

[brv70091-bib-0078] Dunbar, R. I. (1993). Coevolution of neocortical size, group size and language in humans. Behavioral and Brain Sciences 16(4), 681–694.

[brv70091-bib-0079] Easson, C. G. , Slattery, M. , Baker, D. M. & Gochfeld, D. J. (2014). Complex ecological associations: competition and facilitation in a sponge–algal interaction. Marine Ecology Progress Series 507, 153–167.

[brv70091-bib-0080] Elliser, C. R. & Herzing, D. L. (2016). Changes in interspecies association patterns of Atlantic bottlenose dolphins, *Tursiops truncatus*, and Atlantic spotted dolphins, *Stenella frontalis*, after demographic changes related to environmental disturbance. Marine Mammal Science 32(2), 602–618.

[brv70091-bib-0081] Ellison, A. M. , Bank, M. S. , Clinton, B. D. , Colburn, E. A. , Elliott, K. , Ford, C. R. , Foster, D. R. , Kloeppel, B. D. , Knoepp, J. D. , Lovett, G. M. , Mohan, J. , Orwig, D. A. , Rodenhouse, N. L. , Sobczak, W. V. , Stinson, K. A. , *et al*. (2005). Loss of foundation species: consequences for the structure and dynamics of forested ecosystems. Frontiers in Ecology and the Environment 3(9), 479–486.

[brv70091-bib-0082] Espinosa‐Romero, M. J. , Gregr, E. J. , Walters, C. , Christensen, V. & Chan, K. M. (2011). Representing mediating effects and species reintroductions in Ecopath with Ecosim. Ecological Modelling 222(9), 1569–1579.

[brv70091-bib-0083] Essapian, F. S. (1953). The birth and growth of a porpoise. Natural History 62(9), 392–399.

[brv70091-bib-0084] Evans, P. G. H. (1982). Associations between seabirds and cetaceans: a review. Mammal Review 12, 187–206.

[brv70091-bib-0085] Farine, D. R. , Garroway, C. J. & Sheldon, B. C. (2012). Social network analysis of mixed‐species flocks: exploring the structure and evolution of interspecific social behaviour. Animal Behaviour 84(5), 1271–1277.

[brv70091-bib-0086] Feder, H. (1966). Cleaning symbiosis in the marine environment. Symbiosis 1, 327–380.

[brv70091-bib-0087] Fernández‐Cisternas, I. , Majlis, J. , Ávila‐Thieme, M. I. , Lamb, R. W. & Pérez‐Matus, A. (2021). Endemic species dominate reef fish interaction networks on two isolated oceanic islands. Coral Reefs 40(4), 1081–1095.

[brv70091-bib-0088] Ferretti, F. , Worm, B. , Britten, G. L. , Heithaus, M. R. & Lotze, H. K. (2010). Patterns and ecosystem consequences of shark declines in the ocean. Ecology Letters 13, 1055–1071. 20528897 10.1111/j.1461-0248.2010.01489.x

[brv70091-bib-0089] Food and Agriculture Organization of the United Nations (2022). The State of World Fisheries and Aquaculture 2022 (SOFIA). Food & Agriculture Organization of the United Nations (FAO), Rome.

[brv70091-bib-0090] Ford, B. M. & Roberts, J. D. (2019). Evolutionary histories impart structure into marine fish heterospecific co‐occurrence networks. Global Ecology and Biogeography 28(9), 1310–1324.

[brv70091-bib-0091] Foster, S. A. (1985). Group foraging by a coral reef fish: a mechanism for gaining access to defended resources. Animal Behaviour 33(3), 782–792.

[brv70091-bib-0092] Fu, C. , Perry, I. R. , Shin, Y.‐J. , Schweigert, J. , Liu, H. , Viera, D. , Bundy, A. , Fulton, E. A. , Gascuel, D. , Hollowed, A. B. , Mackinson, S. , Marchal, P. , Mullon, C. , Rault, J. , Travaille, K. , *et al*. (2020). The OSMOSE model: representing marine ecosystems as dynamic size spectra. Progress in Oceanography 187, 102395.

[brv70091-bib-0093] Gallo Reynoso, J. P. (1991). Group behavior of common dolphins (*Delphinus delphis*) during prey capture. Anales ‐ Instituto de biologia, Universidad Nacional Autonoma de Mexico. Serie Zoologia 62(2), 253–262.

[brv70091-bib-0094] Garcia, S. M. & Cochrane, K. L. (2005). Ecosystem approach to fisheries: a review of implementation guidelines. ICES Journal of Marine Science 62(3), 311–318.

[brv70091-bib-0095] Gause, G. F. & Witt, A. (1935). Behavior of mixed populations and the problem of natural selection. The American Naturalist 69(725), 596–609.

[brv70091-bib-0096] Gil, M. A. , Emberts, Z. , Jones, H. & St. Mary, C. M. (2017). Social information on fear and food drives animal grouping and fitness. American Naturalist 189(3), 227–241. 10.1086/690055 28221835

[brv70091-bib-0097] Goodale, E. , Beauchamp, G. & Ruxton, G. D. (2017). Mixed‐Species Animal Goups: Behavior, Community Structure, and Conservation. Academic Press, London.

[brv70091-bib-0098] Goodale, E. , Beauchamp, G. , Magrath, R. D. , Nieh, J. C. & Ruxton, G. D. (2010). Interspecific information transfer influences animal community structure. Trends in Ecology & Evolution 25(6), 354–361. 20153073 10.1016/j.tree.2010.01.002

[brv70091-bib-0099] Gostischa, J. , Massolo, A. & Constantine, R. (2021). Multi‐species feeding association dynamics driven by a large generalist predator. Frontiers in Marine Science 8, 1–14.35685121

[brv70091-bib-0100] Gotelli, N. J. , Graves, G. R. & Rahbek, C. (2010). Macroecological signals of species interactions in the Danish avifauna. Proceedings of the National Academy of Sciences 107, 5030–5035. 10.1073/pnas.0914089107 PMC2841898 20194760

[brv70091-bib-0101] Gould, S. J. (1988). Kropotkin was no crackpot. Natural History 97(7), 12–21.

[brv70091-bib-0102] Goyert, H. F. , Gardner, B. , Veit, R. R. , Gilbert, A. T. , Connelly, E. , Duron, M. , Johnson, S. & Williams, K. (2018). Evaluating habitat, prey, and mesopredator associations in a community of marine birds. ICES Journal of Marine Science 75(5), 1602–1612.

[brv70091-bib-0103] Goyert, H. F. , Manne, L. L. & Veit, R. R. (2014). Facilitative interactions among the pelagic community of temperate migratory terns, tunas, and dolphins. Oikos 123(11), 1400–1408.

[brv70091-bib-0104] Grebmeier, J. M. & Harrison, N. M. (1992). Seabird feeding on benthic amphipods facilitated by gray whale activity in the northern Bering Sea. Marine Ecology Progress Series 80, 125–133.

[brv70091-bib-0105] Griffin, D. C. , Harrod, C. , Houghton, J. D. R. & Capellini, I. (2019). Unravelling the macro‐evolutionary ecology of fish–jellyfish associations: life in the ‘gingerbread house’. Proceedings of the Royal Society B: Biological Sciences 286(1902), 20182325. 10.1098/rspb.2018.2325 PMC6452070 30890095

[brv70091-bib-0106] Gruber, J. S. (2010). Key principles of community‐based natural resource management: a synthesis and interpretation of identified effective approaches for managing the commons. Environmental Management 45(1), 52–66. 19083051 10.1007/s00267-008-9235-y

[brv70091-bib-0107] Grutter, A. S. (1997). Effect of the removal of cleaner fish on the abundance and species composition of reef fish. Oecologia 111, 137–143. 28307499 10.1007/s004420050217

[brv70091-bib-0108] Grutter, A. S. (1999). Cleaner fish really do clean. Nature 398(6729), 672–673.

[brv70091-bib-0109] Guse, N. , Markones, N. , Bolduc, F. & Garthe, S. (2013). Distribution of seabirds in the lower estuary and gulf of St. Lawrence (Canada) during summer. Seabird 26, 42–70.

[brv70091-bib-0110] Haak, C. R. , Hui, F. K. , Cowles, G. W. & Danylchuk, A. J. (2020). Positive interspecific associations consistent with social information use shape juvenile fish assemblages. Ecology 101(2), e02920. 31661156 10.1002/ecy.2920

[brv70091-bib-0111] Hager, R. & Jones, C. B. (eds.) (2009). Reproductive Skew in Vertebrates: Proximate and Ultimate Causes. Cambridge University Press, Cambridge.

[brv70091-bib-0112] Hale, K. R. , Valdovinos, F. S. & Martinez, N. D. (2020). Mutualism increases diversity, stability, and function of multiplex networks that integrate pollinators into food webs. Nature Communications 11(1), 2182. 10.1038/s41467-020-15688-w PMC7195475 32358490

[brv70091-bib-0113] Hart, S. P. (2023). How does facilitation influence the outcome of species interactions? Journal of Ecology 111(10), 2094–2104.

[brv70091-bib-0114] Harvey, C. J. (2014). Mediation functions in Ecopath with Ecosim: handle with care. Canadian Journal of Fisheries and Aquatic Sciences 71(7), 1020–1029.

[brv70091-bib-0115] Hatchwell, B. J. (2009). The evolution of cooperative breeding in birds: kinship, dispersal, and life history. Philosophical Transactions of the Royal Society B: Biological Sciences 364(1533), 3217–3227. 10.1098/rstb.2009.0109 PMC2781872 19805429

[brv70091-bib-0116] Hebshi, A. J. , Duffy, D. C. & Hyrenbach, K. D. (2008). Associations between seabirds and subsurface predators around Oahu, Hawaii. Aquatic Biology 4(1), 89–98.

[brv70091-bib-0117] Heise, K. , Barrett‐Lennard, L. G. , Saulitis, E. , Matkin, C. & Bain, D. (2003). Examining the evidence for killer whale predation on Steller sea lions in British Columbia and Alaska. Aquatic Mammals 29(3), 325–334.

[brv70091-bib-0118] Helgason, T. & Gislason, H. (1979). *VPA analysis with species interaction due to predation*. ICES CM 1979/D: 12.

[brv70091-bib-0119] Herzing, D. L. & Johnson, C. (1997). Interspecific interactions between Atlantic spotted dolphins (*Stenella frontalis*) and bottlenose dolphins (*Tursiops truncatus*) in The Bahamas, 1985–1995. Aquatic Mammals 23(2), 85–99.

[brv70091-bib-0120] Hessing, S. , Risser, N. , Pichot, L. , Oudejans, M. G. , Guilpin, M. , Barcelos, L. M. D. , Curé, C. , Hvisser, F. , Kuntz, G. , Kunz, T. K. , Tilmant, L. , Thomas, L. , Leroux, L. , Doré, B. , Macaulay, J. , *et al*. (2024). Context‐driven communication during deep‐sea foraging in a social toothed whale. Royal Society Open Science 11(7), 240558. 39086824 10.1098/rsos.240558 PMC11288665

[brv70091-bib-0121] Hoeksema, J. D. & Bruna, E. M. (2015). Context‐dependent outcomes of mutualistic interactions. In Mutualism (Volume 10, ed J. L. Bronstein ), pp. 181–202. Oxford University Press, Oxford.

[brv70091-bib-0122] Holland, J. N. , DeAngelis, D. L. & Bronstein, J. L. (2002). Population dynamics and mutualism: functional responses of benefits and costs. American Naturalist 159(3), 231–244. 10.1086/338510 18707376

[brv70091-bib-0123] Holmgren, M. & Scheffer, M. (2010). Strong facilitation in mild environments: the stress gradient hypothesis revisited. Journal of Ecology 98(6), 1269–1275.

[brv70091-bib-0124] Holmlund, C. M. & Hammer, M. (1999). Ecosystem services generated by fish populations. Ecological Economics 29(2), 253–268.

[brv70091-bib-0125] Hunter, J. R. , Argue, A. W. , Bayliff, W. H. , Dizon, A. E. , Fonteneau, A. , Goodman, D. , Seckel, G. R. , Fonteneau, A. , Shannon, L. V. , Shingu, C. , Murphy, G. I. , Cayré, P. , Suzuki, Z. , Baylon, J. C. , Sharp, G. D. , Schaefer, K. M. , Ito, R. Y. , Broadhead, G. C. , Draper, R. J. , Perrin, W. F. , *et al*. (1986). The Dynamics of Tuna Movements: An Evaluation of Past and Future Research. Food and Agriculture Organization of the United Nations, Rome.

[brv70091-bib-0126] Hurtubise, J. (2016). Evolution of Subsistence and Commercial Inuit Fisheries in the Territory of Nunavut, Canada: Research and Summation of Landings, Quotas, Gear Type, Significance, Use, and Status of Hunted Marine Species (Marine Affairs Program Technical Report No. 14). Dalhousie University, Marine Affairs Program, Halifax.

[brv70091-bib-0127] Inagaki, K. Y. , Mendes, T. C. , Quimbayo, J. P. , Cantor, M. & Sazima, I. (2020). The structure of fish follower‐feeding associations at three oceanic islands in southwestern Atlantic. Environmental Biology of Fishes 103, 1–11.

[brv70091-bib-0128] International Union for Conservation of Nature (2024). IUCN Red List of Threatened Species . https://www.iucnredlist.org.

[brv70091-bib-0129] Jensen, A. M. , Sheehan, G. W. & MacLean, S. A. (2009). Inuit and marine mammals. In Encyclopedia of Marine Mammals, Second Edition (eds W. F. Perrin , B. Würsig and J. G. M. Thewissen ), pp. 628–637. Academic Press, San Diego.

[brv70091-bib-0130] Jones, C. G. , Lawton, J. H. & Shachak, M. (1994). Organisms as ecosystem engineers. Oikos 69(3), 373–386.

[brv70091-bib-0131] Jones, C. G. , Lawton, J. H. & Shachak, M. (1997). Positive and negative effects of organisms as physical ecosystem engineers. Ecology 78(7), 1946–1957.

[brv70091-bib-0132] Jourdain, E. & Vongraven, D. (2017). Humpback whale (*Megaptera novaeangliae*) and killer whale (*Orcinus orca*) feeding aggregations for foraging on herring (*Clupea harengus*) in northern Norway. Mammalian Biology 86(September), 27–32.

[brv70091-bib-0133] Jurado‐Molina, J. , Livingston, P. A. & Ianelli, J. N. (2005). Incorporating predation interactions in a statistical catch‐at‐age model for Gulf of Alaska walleye pollock (*Theragra chalcogramma*). Canadian Journal of Fisheries and Aquatic Sciences 62(9), 1865–1874.

[brv70091-bib-0134] Karban, R. , Grof‐Tisza, P. & Holyoak, M. (2012). Facilitation of tiger moths by outbreaking tussock moths that share the same host plants. Journal of Animal Ecology 81(5), 1095–1102. 22553976 10.1111/j.1365-2656.2012.01993.x

[brv70091-bib-0135] Kawai, T. & Tokeshi, M. (2007). Testing the facilitation–competition paradigm under the stress‐gradient hypothesis: decoupling multiple stress factors. Proceedings of the Royal Society B: Biological Sciences 274(1624), 2503–2508. 10.1098/rspb.2007.0871 PMC2274984 17686725

[brv70091-bib-0136] Kéfi, S. , Berlow, E. L. , Wieters, E. A. , Joppa, L. N. , Wood, S. A. , Brose, U. , Navarrete, S. A. , Blanchard, J. L. , Flores, J. D. , Jara, F. , Lopez, B. C. , Moreno, C. A. , Ramos‐Jiliberto, R. , Valdivia, N. , Stone, L. , *et al*. (2015). Network structure beyond food webs: mapping non‐trophic and trophic interactions on Chilean rocky shores. Ecology 96(1), 291–303. 26236914 10.1890/13-1424.1

[brv70091-bib-0137] Kéfi, S. , Miele, V. , Wieters, E. A. , Navarrete, S. A. & Berlow, E. L. (2016). How structured is the entangled bank? The surprisingly simple organization of multiplex ecological networks leads to increased persistence and resilience. PLoS Biology 14(8), e1002527. 27487303 10.1371/journal.pbio.1002527 PMC4972357

[brv70091-bib-0138] Kiszka, J. , Perrin, W. F. , Pusineri, C. & Ridoux, V. (2011). What drives Island‐associated tropical dolphins to form mixed‐species associations in the southwest Indian Ocean? Journal of Mammalogy 92(5), 1105–1111.

[brv70091-bib-0139] Koen‐Alonso, M. & Yodzis, P. (2005). Multispecies modelling of some components of the marine community of northern and central Patagonia, Argentina. Canadian Journal of Fisheries and Aquatic Sciences 62(7), 1490–1512.

[brv70091-bib-0140] Kompas, T. , Grafton, R. Q. & Che, T. N. (2010). Bioeconomic losses from overharvesting tuna. Conservation Letters 3(3), 177–183.

[brv70091-bib-0141] Krause, J. , Croft, D. P. & James, R. (2007). Social network theory in the behavioural sciences: potential applications. Behavioral Ecology and Sociobiology 62, 15–27. 32214613 10.1007/s00265-007-0445-8 PMC7079911

[brv70091-bib-0142] Krause, J. , James, R. , Franks, D. W. & Croft, D. P. (2015). Animal Social Networks. Oxford University Press, Oxford.

[brv70091-bib-0143] Kropotkin, P. (1902). Mutual Aid: a Factor of Evolution. McClure, Phillips & Co, London.

[brv70091-bib-0144] Lafferty, K. D. & Suchanek, T. H. (2016). Revisiting Paine's 1966 sea star removal experiment, the most‐cited empirical article in the American naturalist. The American Naturalist 188(4), 365–378. 10.1086/688045 27622872

[brv70091-bib-0145] Lang, S. D. & Farine, D. R. (2017). A multidimensional framework for studying social predation strategies. Nature Ecology & Evolution 1(9), 1230–1239. 29046557 10.1038/s41559-017-0245-0

[brv70091-bib-0146] Le Bagousse‐Pinguet, Y. , Xiao, S. , Brooker, R. W. , Gross, N. , Liancourt, P. , Straile, D. , Michalzik, B. , Baguette, M. , Rixen, C. , Schöb, C. , Spiegelberger, T. , Boldoch, A. , Leps, J. , Maaestri, E. , *et al*. (2014). Facilitation displaces hotspots of diversity and allows communities to persist in heavily stressed and disturbed environments. Journal of Vegetation Science 25(1), 66–76.

[brv70091-bib-0147] Lett, C. , Semeria, M. , Thiebault, A. & Tremblay, Y. (2014). Effects of successive predator attacks on prey aggregations. Theoretical Ecology 7, 239–252. 10.1111/1365-2656.12455 26768335

[brv70091-bib-0148] Li, X. , Zhong, Z. , Sanders, D. , Smit, C. , Wang, D. , Nummi, P. , Zhang, Z. , Jia, Z. , Li, Y. , Chen, Y. , Liu, Q. , Xu, H. , Zhang, H. , Han, X. , *et al*. (2018). Reciprocal facilitation between large herbivores and ants in a semi‐arid grassland. Proceedings of the Royal Society B 285(1888), 20181665. 30305439 10.1098/rspb.2018.1665 PMC6191696

[brv70091-bib-0149] Limbaugh, C. (1961). Cleaning symbiosis. Scientific American 205(2), 42–49.

[brv70091-bib-0150] Lin, Y. , Berger, U. , Grimm, V. & Ji, Q. R. (2012). Differences between symmetric and asymmetric facilitation matter: exploring the interplay between modes of positive and negative plant interactions. Journal of Ecology 100(6), 1482–1491.

[brv70091-bib-0151] Lin, Y. , Berger, U. , Yue, M. & Grimm, V. (2016). Asymmetric facilitation can reduce size inequality in plant populations resulting in delayed density‐dependent mortality. Oikos 125(8), 1153–1161.

[brv70091-bib-0152] Lindenmayer, D. & Burgman, M. (2005). Practical Conservation Biology. CSIRO Publishing, Collingwood.

[brv70091-bib-0153] Link, J. S. (2010). Ecosystem‐Based Fisheries Management: Confronting Tradeoffs. Cambridge University Press, Cambridge.

[brv70091-bib-0154] Long, R. D. , Charles, A. & Stephenson, R. L. (2015). Key principles of marine ecosystem‐based management. Marine Policy 57, 53–60.

[brv70091-bib-0155] Lukoschek, V. & McCormick, M. I. (2000). A review of multispecies foraging associations in fishes and their ecological significance. Proceedings of the Ninth International Coral Reef Symposium 1, 467–474.

[brv70091-bib-0156] Machado, A. M. , Daura‐Jorge, F. G. , Herbst, D. F. , Simões‐Lopes, P. C. , Ingram, S. N. , De Castilho, P. V. & Peroni, N. (2019). Artisanal fishers' perceptions of the ecosystem services derived from a dolphin‐human cooperative fishing interaction in southern Brazil. Ocean & Coastal Management 173, 148–156.

[brv70091-bib-0157] Malanson, G. P. & Resler, L. M. (2016). A size‐gradient hypothesis for alpine treeline ecotones. Journal of Mountain Science 13, 1154–1161.

[brv70091-bib-0158] McDonald, A. D. , Little, L. R. , Gray, R. , Fulton, E. A. , Sainsbury, K. J. & Lyne, V. D. (2008). An agent‐based modelling approach to evaluation of multiple‐use management strategies for coastal marine ecosystems. Mathematics and Computers in Simulation 78(2), 401–411.

[brv70091-bib-0159] McInnes, J. D. , Lester, K. M. , Dill, L. M. , Mathieson, C. R. , West‐Stap, P. J. , Marcos, S. L. , Trites, A. W. , Ford, J. K. B. , Pilkington, J. F. , Pilkington, G. A. , Stredulins, M. , Tremblay, Y. , Jones, M. C. , Schall, E. , Ramsey, A. R. , *et al*. (2024). Foraging behaviour and ecology of transient killer whales within a deep submarine canyon system. PLoS One 19(3), e0299291. 38507673 10.1371/journal.pone.0299291 PMC10954312

[brv70091-bib-0160] McIntire, E. J. & Fajardo, A. (2014). Facilitation as a ubiquitous driver of biodiversity. New Phytologist 201(2), 403–416. 24102266 10.1111/nph.12478

[brv70091-bib-0161] Michalet, R. , Brooker, R. W. , Cavieres, L. A. , Kikvidze, Z. , Lortie, C. J. , Pugnaire, F. I. , Valiente‐Banuet, A. , Callaway, R. M. , Armas, C. , Maestre, F. T. , Holzapfel, C. , Monserrud, R. A. , Lavergne, S. , Rebertus, A. J. , Shipley, B. , *et al*. (2006). Do biotic interactions shape both sides of the humped‐back model of species richness in plant communities? Ecology Letters 9(7), 767–773. 16796565 10.1111/j.1461-0248.2006.00935.x

[brv70091-bib-0162] Michalet, R. , Le Bagousse‐Pinguet, Y. , Maalouf, J. P. & Lortie, C. J. (2014). Two alternatives to the stress‐gradient hypothesis at the edge of life: the collapse of facilitation and the switch from facilitation to competition. Journal of Vegetation Science 25(2), 609–613.

[brv70091-bib-0163] Michalet, R. & Pugnaire, F. I. (2016). Facilitation in communities: underlying mechanisms, community, and ecosystem implications. Functional Ecology 30(1), 3–9.

[brv70091-bib-0164] Monier, S. A. , Veit, R. R. & Manne, L. L. (2020). Changes in positive associations among vertebrate predators at South Georgia during winter. Polar Biology 43(10), 1439–1451.

[brv70091-bib-0165] Mukherjee, I. & Bhat, A. (2023). What drives mixed‐species shoaling among wild zebrafish? The roles of predators, food access, abundance of conspecifics and familiarity. Journal of Experimental Biology 226(5), jeb244644. 10.1242/bio.059529 PMC9915908 36583380

[brv70091-bib-0166] Munn, C. A. & Terborgh, J. W. (1979). Multi‐species territoriality in neotropical foraging flocks. The Condor 81(4), 338–347.

[brv70091-bib-0167] Norris, K. S. & Dohl, T. (1980). Behavior of the Hawaiian spinner dolphin, Stenella longirostris. Fishery Bulletin 77(4), 821–849.

[brv70091-bib-0168] Norris, K. S. & Schilt, C. R. (1988). Cooperative societies in three‐dimensional space: On the origins of aggregations, flocks, and schools, with special reference to dolphins and fish. Ethology and Sociobiology 9(2‐4), 149–179.

[brv70091-bib-0169] Olson, R. J. & Watters, G. M. (2003). A model of the pelagic ecosystem in the eastern tropical Pacific Ocean. Inter‐American Tropical Tuna Commission Bulletin 22, 133–218.

[brv70091-bib-0170] Ormond, R. F. (1980). Aggressive mimicry and other interspecific feeding associations among Red Sea coral reef predators. Journal of Zoology 191(2), 247–262.

[brv70091-bib-0171] Paijmans, K. C. S. , Reardon, E. E. & Ward, A. J. W. (2020). Predation avoidance and foraging efficiency contribute to mixed‐species shoaling by tropical and temperate fishes. Behavioral Ecology and Sociobiology 74, 21. 10.1111/jfb.14277 32031243

[brv70091-bib-0172] Paine, R. T. (1966). Food web complexity and species diversity. The American Naturalist 100(910), 65–75.

[brv70091-bib-0173] Peterson, D. , Hanazaki, N. & Simões‐Lopes, P. C. (2008). Natural resource appropriation in cooperative artisanal fishing between fishermen and dolphins (*Tursiops truncatus*) in Laguna, Brazil. Ocean and Coastal Management 51(6), 469–475.

[brv70091-bib-0174] Pierotti, R. (1988). Interactions between marine birds and mammals in the Northwest Atlantic Ocean. Ecology 69(4), 1278–1285.

[brv70091-bib-0175] Pitcher, T. J. (2012). The Behaviour of Teleost Fishes. Springer, Dordrecht.

[brv70091-bib-0176] Pitman, R. L. & Ballance, L. T. (1992). Parkinson's petrel distribution and foraging ecology in the eastern Pacific: aspects of an exclusive feeding relationship with dolphins. The Condor 94(4), 825–835.

[brv70091-bib-0177] Pitman, R. L. , Deecke, V. B. , Gabriele, C. M. , Srinivasan, M. , Black, N. , Denkinger, J. , Durban, J. W. , Matkin, C. O. , Matkin, D. R. , Perry, S. L. , Robbins, J. , Santos, M. B. , Spitz, J. , Ternullo, R. , Townsend, F. I. , *et al*. (2017). Humpback whales interfering when mammal‐eating killer whales attack other species: mobbing behavior and interspecific altruism? Marine Mammal Science 33(1), 7–58.

[brv70091-bib-0178] Plagányi, É. E. (2007). Models for an Ecosystem Approach to Fisheries (FAO Fisheries Technical Paper No. 477). Food and Agriculture Organization of the United Nations, Rome.

[brv70091-bib-0179] Plummer, M. L. , Harvey, C. J. , Anderson, L. E. , Guerry, A. D. & Ruckelshaus, M. H. (2013). The role of eelgrass in marine community interactions and ecosystem services: results from ecosystem‐scale food web models. Ecosystems 16(2), 237–251.

[brv70091-bib-0180] Polis, G. A. , Anderson, W. B. & Holt, R. D. (1997). Toward an integration of landscape and food web ecology: the dynamics of spatially subsidized food webs. Annual Review of Ecology and Systematics 28, 289–316.

[brv70091-bib-0181] Polovina, J. J. (1984). Model of a coral reef ecosystem. I. The Ecopath model and its application to French frigate shoals. Coral Reefs 3(1), 1–11.

[brv70091-bib-0182] Pope, J. G. (1979). A modified cohort analysis in which constant mortality is replaced by estimates of predation levels. In ICES CM Documents, 1979/H:16, pp. 1–12.

[brv70091-bib-0183] Pope, J. G. (1991). The ICES multispecies assessment working group: evolution, insights, and future problems. ICES Marine Science Symposia 193, 22–33.

[brv70091-bib-0184] Pope, J. G. & Macer, C. T. (1991). Multispecies virtual population analysis of North Sea cod, haddock, and whiting, 1963–1987. ICES Marine Science Symposia 193, 12–21.

[brv70091-bib-0185] Pott, C. & Wiedenfeld, D. A. (2017). Information gaps limit our understanding of seabird bycatch in global fisheries. Biological Conservation 210, 192–204.

[brv70091-bib-0186] Pöysä, H. (1992). Group foraging in patchy environments: the importance of coarse‐level local enhancement. Ornis Scandinavica 23, 159–166.

[brv70091-bib-0187] Pullin, A. S. (2002). Conservation Biology. Cambridge University Press, Cambridge.

[brv70091-bib-0188] Quimbayo, J. P. , Cantor, M. , Dias, M. S. , Moura, R. L. , França, L. M. , Ledesma, M. , Morante, F. A. , Bellwood, D. R. , Coll, M. , Freitas, M. O. , Floeter, S. R. , Pereira, P. H. C. , Nunes, L. T. , Vasconcelos, R. , Longo, G. O. , *et al*. (2018). The global structure of marine cleaning mutualistic networks. Global Ecology and Biogeography 27, 1238–1250.

[brv70091-bib-0189] Quimbayo, J. P. , Freitas, R. , Rocha, L. A. & Pinheiro, H. T. (2023). Are cleaning interactions offered by large cleaners positive? Journal of Natural History 57(17–20), 1152–1156.

[brv70091-bib-0190] Ramos, J. A. (2000). Characteristics of foraging habitats and chick food provisioning by tropical roseate terns. The Condor 102(4), 795–803.

[brv70091-bib-0191] Rogan, E. & Mackey, M. (2007). Megafauna bycatch in drift nets for albacore tuna (*Thunnus alalunga*) in the NE Atlantic. Fisheries Research 86(1), 6–14.

[brv70091-bib-0192] Roman, J. & McCarthy, J. J. (2010). The whale pump: marine mammals enhance primary productivity in a coastal basin. PLoS One 5(10), e13255. 20949007 10.1371/journal.pone.0013255 PMC2952594

[brv70091-bib-0193] Santos, M. L. , Lemos, V. M. & Vieira, J. P. (2018). No mullet, no gain: cooperation between dolphins and cast net fishermen in southern Brazil. Zoologia (Curitiba) 35, e24446.

[brv70091-bib-0194] Santos‐Silva, B. , Hanazaki, N. , Daura‐Jorge, F. G. & Cantor, M. (2022). Social foraging can benefit artisanal fishers who interact with wild dolphins. Behavioral Ecology and Sociobiology 76(3), 42.

[brv70091-bib-0195] Sazima, I. , Krajewski, J. P. , Bonaldo, R. M. & Sazima, C. (2007). The goatfish *Pseudupeneus maculatus* and its follower fishes at an oceanic Island in the tropical west Atlantic. Journal of Fish Biology 70(3), 758–771.

[brv70091-bib-0196] Scott, M. D. , Chivers, S. J. , Olson, R. J. , Fiedler, P. C. & Holland, K. (2012). Pelagic predator associations: tuna and dolphins in the eastern tropical Pacific Ocean. Marine Ecology Progress Series 458, 283–302.

[brv70091-bib-0197] Silknetter, S. , Creed, R. P. , Brown, B. L. , Frimpong, E. A. , Skelton, J. & Peoples, B. K. (2020). Positive biotic interactions in freshwaters: a review and research directive. Freshwater Biology 65(4), 811–832.

[brv70091-bib-0198] Silverman, E. , Veit, R. & Nevitt, G. (2004). Nearest neighbors as foraging cues: information transfer in a patchy environment. Marine Ecology Progress Series 277, 25–36.

[brv70091-bib-0199] Simões‐Lopes, P. C. (1998). Intraspecific agonistic behavior of *Tursiops truncatus* (Cetacea, Delphinidae) during dolphin‐human cooperative fishing in southern Brazil. Biotemas 11(2), 165–171.

[brv70091-bib-0200] Smith, B. D. , Tun, M. T. , Chit, A. M. , Win, H. & Moe, T. (2009). Catch composition and conservation management of a human–dolphin cooperative cast‐net fishery in the Ayeyarwady River, Myanmar. Biological Conservation 142(5), 1042–1049.

[brv70091-bib-0201] Smith, J. E. (2014). Hamilton's legacy: kinship, cooperation, and social tolerance in mammalian groups. Animal Behaviour 92, 291–304.

[brv70091-bib-0202] Soares, M. C. , Bshary, R. , Cardoso, S. C. & Côté, I. M. (2008a). Does competition for clients increase service quality in cleaning gobies? Ethology 114(12), 1173–1181.

[brv70091-bib-0203] Soares, M. C. , Côté, I. M. , Cardoso, S. & Bshary, R. (2008b). The cleaning goby mutualism: a system without punishment, partner switching or tactile stimulation. Journal of Zoology 276(3), 306–312.

[brv70091-bib-0204] Soares, M. C. , Oliveira, R. F. , Ros, A. F. H. & Bshary, R. (2012). Tactile stimulation lowers stress in fish. Nature Communications 2, 534. 10.1038/ncomms1547 22086335

[brv70091-bib-0205] Sparre, P. (1993). Introduction to multispecies virtual population analysis. ICES Marine Science Symposia 193, 12–21.

[brv70091-bib-0206] Spottiswoode, C. N. , Begg, K. S. & Begg, C. M. (2016). Reciprocal signaling in honeyguide‐human mutualism. Science 353(6297), 387–389. 27463674 10.1126/science.aaf4885

[brv70091-bib-0207] Sridhar, H. , Beauchamp, G. & Shanker, K. (2009). Why do birds participate in mixed‐species foraging flocks? A large‐scale synthesis. Animal Behaviour 78(2), 337–347.

[brv70091-bib-0208] Stachowicz, J. J. (2001). Mutualism, facilitation, and the structure of ecological communities. Bioscience 51(3), 235–246.

[brv70091-bib-0209] Stachowicz, J. J. & Hay, M. E. (1999). Reducing predation through chemically mediated camouflage: indirect effects of plant defenses on herbivores. Ecology 80(2), 495–509.

[brv70091-bib-0210] Stensland, E. , Angerbjörn, A. & Berggren, P. (2003). Mixed species groups in mammals. Mammal Review 33(3–4), 205–223.

[brv70091-bib-0211] Strand, S. (1988). Following behavior: interspecific foraging associations among Gulf of California reef fishes. Copeia 2, 351–357.

[brv70091-bib-0212] Svenning, J. C. , Gravel, D. , Holt, R. D. , Schurr, F. M. , Thuiller, W. , Münkemüller, T. , Harpke, A. , Pyšek, P. , Normand, S. , Hultén, J. , Kissling, W. D. , Lenoir, J. , Madelenat, M. , Müller‐Scharer, H. , Tronstad, L. , *et al*. (2014). The influence of interspecific interactions on species range expansion rates. Ecography 37(12), 1198–1209. 25722537 10.1111/j.1600-0587.2013.00574.x PMC4338500

[brv70091-bib-0213] Syme, J. , Kiszka, J. J. & Parra, G. J. (2021). Dynamics of cetacean mixed‐species groups: a review and conceptual framework for assessing their functional significance. Frontiers in Marine Science 8(June), 1–19.35685121

[brv70091-bib-0214] Syme, J. , Kiszka, J. J. & Parra, G. J. (2023). Multiple social benefits drive the formation of mixed‐species groups of Australian humpback and Indo‐Pacific bottlenose dolphins. Behavioral Ecology and Sociobiology 77(4), 1–16.

[brv70091-bib-0215] Tews, J. , Brose, U. , Grimm, V. , Tielbörger, K. , Wichmann, M. C. , Schwager, M. & Jeltsch, F. (2004). Animal species diversity driven by habitat heterogeneity/diversity: the importance of keystone structures. Journal of Biogeography 31(1), 79–92.

[brv70091-bib-0216] Thiebault, A. , Mullers, R. H. , Pistorius, P. A. & Tremblay, Y. (2014). Local enhancement in a seabird: reaction distances and foraging consequence of predator aggregations. Behavioral Ecology 25(6), 1302–1310.

[brv70091-bib-0217] Thiebault, A. , Semeria, M. , Lett, C. & Tremblay, Y. (2016). How to capture fish in a school? Effect of successive predator attacks on seabird feeding success. Journal of Animal Ecology 85(1), 157–167. 26768335 10.1111/1365-2656.12455

[brv70091-bib-0218] Thiebot, J. B. & Weimerskirch, H. (2013). Contrasted associations between seabirds and marine mammals across four biomes of the southern Indian Ocean. Journal of Animal Ecology 82(3), 674–685.

[brv70091-bib-0219] Thompson, L. M. (2010). Long‐Term Inter‐ and Intra‐Species Interactions of Marine Tucuxi (Sotalia guianensis) and Common Bottlenose (Tursiops truncatus) dolphins in Gandoca‐Manzanillo, Costa Rica. Master’s Thesis: Hofstra University, Hempstead, New York.

[brv70091-bib-0220] Thomsen, M. S. & South, P. M. (2019). Communities and attachment networks associated with primary, secondary and alternative foundation species: a case of stressed and disturbed stands of southern bull kelp. Diversity 11(4), 56.

[brv70091-bib-0221] Thomsen, M. S. & Wernberg, T. (2014). On the generality of cascading habitat‐formation. Proceedings of the Royal Society B: Biological Sciences 281(1777), 20131994. 10.1098/rspb.2013.1994 PMC3896006 24403322

[brv70091-bib-0222] Thomsen, M. S. , Wernberg, T. , Altieri, A. , Tuya, F. , Gulbransen, D. , McGlathery, K. J. , Holmer, M. & Silliman, B. R. (2010). Habitat cascades: the conceptual context and global relevance of facilitation cascades via habitat formation and modification. Integrative and Comparative Biology 50(2), 158–175. 21558196 10.1093/icb/icq042

[brv70091-bib-0223] Todes, D. P. (1987). Darwin's Malthusian metaphor and Russian evolutionary thought, 1859‐1917. Isis 78(4), 537–551. 3329160 10.1086/354551

[brv70091-bib-0224] Townsend, S. (2021). A systematic review of facilitation in intertidal habitats. Master's project, Nicholas School of the Environment, Duke University, USA.

[brv70091-bib-0225] Trenkel, V. M. , Skaug, H. J. & Berg, C. W. (2004). An overview of the GADGET modelling framework for fisheries. ICES Journal of Marine Science 61(3), 348–356.

[brv70091-bib-0226] Trillmich, F. (1996). Parental investment in pinnipeds. In Behavioural Ecology: An Evolutionary Approach. Fourth Edition (eds J. R. Krebs and N. B. Davies ), pp. 69–97. Blackwell, Oxford.

[brv70091-bib-0227] van der Wal, J. E. M. , Spottiswoode, C. N. , Uomini, N. , Cantor, M. , Daura‐Jorge, F. G. , Afan, A. I. , Attwood, M. C. , Amphaeris, J. , Balasani, F. , Begg, C. M. , Blair, C. J. , Bronstein, J. L. , Buanachique, I. O. , Cuthill, R. R. T. , Das, J. , *et al*. (2022). Safeguarding human–wildlife cooperation. Conservation Letters 15(4), 1–18. 10.1111/conl.12886 PMC9540276 36248252

[brv70091-bib-0228] Vaughan, D. B. , Grutter, A. S. , Costello, M. J. & Hutson, K. S. (2017). Cleaner fishes and shrimp diversity and a re‐evaluation of cleaning symbioses. Fish and Fisheries 18(4), 698–716.

[brv70091-bib-0229] Veit, R. R. & Harrison, N. M. (2017). Positive interactions among foraging seabirds, marine mammals and fishes and implications for their conservation. Frontiers in Ecology and Evolution 5, 1–8.

[brv70091-bib-0230] Verwijmeren, M. , Rietkerk, M. , Wassen, M. J. & Smit, C. (2013). Interspecific facilitation and critical transitions in arid ecosystems. Oikos 122(3), 341–347.

[brv70091-bib-0231] Wade, P. R. , Long, K. J. , Francis, T. B. , Punt, A. E. , Hammond, P. S. , Heinemann, D. , Barlow, J. , Carretta, J. V. , Curry, B. E. , Harwood, J. , Hobbs, R. C. , Moore, J. E. , Petersen, S. L. , Read, A. J. , Reeves, R. R. , *et al*. (2021). Best practices for assessing and managing bycatch of marine mammals. Frontiers in Marine Science 8(November), 1–19.35685121

[brv70091-bib-0232] Waldie, P. A. , Blomberg, S. P. , Cheney, K. L. , Goldizen, A. W. & Grutter, A. S. (2011). Long‐term effects of the cleaner fish *Labroides dimidiatus* on coral reef fish communities. PLoS One 6(6), e21201. 21731670 10.1371/journal.pone.0021201 PMC3123342

[brv70091-bib-0233] Walters, C. J. , Christensen, V. , Martell, S. J. & Kitchell, J. F. (2005). Possible ecosystem impacts of applying MSY policies from single‐species assessment. ICES Journal of Marine Science 62(3), 558–568.

[brv70091-bib-0234] Walters, C. , Christensen, V. & Pauly, D. (1997). Structuring dynamic models of exploited ecosystems from trophic mass‐balance assessments. Reviews in Fish Biology and Fisheries 7, 139–172.

[brv70091-bib-0235] White, C. F. , Pratt, H. L. , Pratt, T. C. & Whitney, N. M. (2022). Interspecific foraging association of a nurse shark (*Ginglymostoma cirratum*) with bottlenose dolphins (*Tursiops truncatus*). Animal Biotelemetry 10(1), 1–7.

[brv70091-bib-0236] Witman, J. D. (1987). Subtidal coexistence: storms, grazing, mutualism, and the zonation of kelps and mussels. Ecological Monographs 57(2), 167–187.

[brv70091-bib-0237] Wolf, N. G. (1987). Schooling tendency and foraging benefit in the ocean surgeonfish. Behavioral Ecology and Sociobiology 21, 59–63.

[brv70091-bib-0238] Woodworth‐Jefcoats, P. A. , Blanchard, J. L. & Drazen, J. C. (2019). Size‐based ecosystem modeling: linking ecosystem structure and function. Ecological Modelling 392, 1–17.

[brv70091-bib-0239] Wooster, E. I. , Gaynor, K. M. , Carthey, A. J. , Wallach, A. D. , Stanton, L. A. , Ramp, D. & Lundgreen, E. J. (2023). Animal cognition and culture mediate predator–prey interactions. Trends in Ecology & Evolution 38(8), 707–719. 10.1016/j.tree.2023.09.012 37839906

[brv70091-bib-0240] Xiao, S. , Michalet, R. , Wang, G. & Chen, S. Y. (2009). The interplay between species' positive and negative interactions shapes the community biomass–species richness relationship. Oikos 118(9), 1343–1348.

[brv70091-bib-0241] Yodzis, P. (1998). Local trophodynamics and the interaction of marine mammals and fisheries in the Benguela ecosystem. Journal of Animal Ecology 67(4), 635–658.

[brv70091-bib-0242] Yodzis, P. & Innes, S. (1992). Body size and consumer–resource dynamics. American Naturalist 139(6), 1151–1175.

[brv70091-bib-0243] Zepeda, V. & Martorell, C. (2019). Seed mass equalises the strength of positive and negative plant–plant interactions in a semi‐arid grassland. Oecologia 190(2), 287–296. 30662998 10.1007/s00442-018-04326-4

[brv70091-bib-0244] Žydelis, R. , Small, C. & French, G. (2013). The incidental catch of seabirds in gillnet fisheries: a global review. Biological Conservation 162, 76–88.

